# Existing Evidence from Economic Evaluations of Antimicrobial Resistance—A Systematic Literature Review

**DOI:** 10.3390/antibiotics14111072

**Published:** 2025-10-24

**Authors:** Sajan Gunarathna, Yongha Hwang, Jung-Seok Lee

**Affiliations:** International Vaccine Institute, Seoul 08826, Republic of Korea; sajan.gunarathna@ivi.int (S.G.); yongha.hwang@ivi.int (Y.H.)

**Keywords:** antimicrobial resistance, cost effectiveness, cost of illness, economic evaluations, limitations

## Abstract

**Background/Objectives**: Although antimicrobial resistance (AMR) is recognized as a critical global health threat across human, animal, and environmental domains, evidence from AMR economic evaluations remains limited. This study systematically reviewed available studies, emphasizing existing evidence and reported limitations in AMR-related economic evaluations. **Methods**: A comprehensive review of peer-reviewed empirical studies was conducted, including publications up to July 2023 without temporal restrictions, but limited to English-language articles. Literature searches were undertaken in PubMed and Cochrane using a search strategy centered on the terms “economic evaluations” and “antimicrobial resistance.” Screening and data extraction were performed by two reviewers independently, with disagreements resolved through consensus or consultation with a third reviewer. Findings were synthesized narratively. **Results**: Of the 3682 records screened, 93 studies were included. Evidence gaps were identified across income and geographic regions, particularly in low- and middle-income countries (LMICs) and the African, Southeast Asian, and Eastern Mediterranean regions. Studies were comparatively more numerous in high-income countries (HICs) and the European and Americas regions. Substantial gaps also existed in one health approach and community-based evaluations. Nine major study limitations were identified, with many interlinked. The most frequent issues included limited generalizability primarily due to inadequate sampling approaches (*n* = 16), and single-center studies (*n* = 11), alongside errors in cost estimation (*n* = 4), and lack of consideration for essential features or information (*n* = 3). **Conclusions**: The review highlights persistent evidence gaps and recurring methodological shortcomings in AMR economic evaluations. Addressing these limitations, particularly in LMICs, will strengthen the evidence base and better inform policy implementation to combat AMR effectively.

## 1. Background

Antimicrobial drugs, which include antibiotics, antivirals, antifungals, and antiparasitics, serve as essential medicines for preventing and treating infectious diseases in humans, animals, and plants [1]. Despite these agents’ life-saving impact over the years, antimicrobial resistance (AMR) is a significant global health challenge and is defined as the ability of microorganisms to counteract the action of antimicrobial agents, particularly when antibiotics lose their effectiveness in inhibiting bacterial growth [2]. The major drivers of AMR are excessive use and inappropriate prescription practices, including incorrect drug selection, duration, and frequency [3].

The World Health Organization (WHO) projected that the unrelenting overuse of antibiotics could result in 10 million global deaths by 2050 and may lead to 24 million people experiencing extreme poverty by 2030 [4]. Additionally, the World Bank (WB) estimated that AMR could result in USD 1 trillion in additional healthcare costs by 2050 and up to USD 3.4 trillion in gross domestic product losses per year by 2030 [5]. Furthermore, a total annual health sector cost of USD 28.2 million for methicillin-resistant *Staphylococcus aureus* in Canada was reported in 1998 [6], and a total annual hospital cost of USD 5.2 million for Ceftriaxone-resistant *Escherichia coli* bloodstream infections in Australia was reported in 2014 [7]. AMR has become a primary health concern globally because of its life-threatening impact and substantial economic burden. A recent systematic review reported that the global burden of AMR was due mainly to the pathogens *Escherichia coli*, *Acinetobacter baumannii*, *Klebsiella pneumoniae*, *Salmonella* spp. and *Staphylococcus aureus* [8]. This has elevated AMR to a critical global policy issue, and the current focus is to reduce its use, even though antibiotics are indispensable for human, animal, and plant health [9,10].

In this context, AMR evaluation studies are critical for addressing this policy issue by generating evidence on the burden of AMR and highlighting its severity. However, a review article highlighted that the prevailing studies are generally inadequate because of methodological gaps, including narrow perspectives of the analysis and problems associated with the input-cost data [11]. Good-quality research, including economic evaluations and comprehensive modeling, is needed to determine the most effective strategies to combat AMR and to find ways to address these methodological difficulties. More importantly, the number of economic evaluations of AMR is limited globally; moreover, existing evidence is disproportionately lower in LMICs.

With this background, it is critical to understand what evidence is currently available, and a review reporting the economic evidence and limitations of each published study of AMR-related economic evaluations would aid in designing and implementing policy interventions, ultimately helping combat AMR. Therefore, the main aim of this review is to report existing evidence and limitations in published AMR-related economic evaluations worldwide.

## 2. Methods

### 2.1. Study Design

We employed the methodology of systematic literature review, following the principles of the ‘Preferred Reporting Items for Systematic Reviews and Meta-Analyses’ (PRISMA) guidelines. The PRISMA Abstract Checklist and the full PRISMA Checklist are presented in Supplementary File S1 and Supplementary File S2, respectively.

### 2.2. Eligibility Criteria

All peer-reviewed primary empirical studies on economic evaluations of AMR were assessed. There were no temporal restrictions for publications; studies published up to July 2023 were considered. The searches were limited only to the English-language publications.

### 2.3. Search Strategy

Data sources: The electronic search included bibliographic database searches via PubMed and Cochrane Library. The search was performed on 1 July 2023.

Search terms: The search strategy consisted of two high-level categories, namely, ‘economic evaluations’ and ‘antimicrobial resistance,’ which are searched via medical subject headings (MeSH) and text words. Each of the high-level categories included specific search terms, and the two high-level categories were combined as shown below:

[(“antimicrobial*” OR “antibiotic*” OR “antibacterial*” OR “antiviral*” OR “antifungal*” OR “antiparasitic*”) AND (“resistan*” OR “overus*” OR “misus*”) AND (“economic evaluation” OR “economic burden” OR “cost effective*” OR “cost utility” OR “cost benefit” OR “cost of illness” OR “cost minimization” OR “incremental cost-effectiveness ratio” OR “QALY*” OR “quality adjusted life year*” OR “DALY*” OR “disability adjusted life year*”)]

### 2.4. Selecting Studies

The outcomes of the searches in each database were exported and deduplicated using Mendeley Desktop 1.19.8 reference management software. Each study’s eligibility was determined based on the basis of the respective study title and abstract (and keywords where applicable) screening. Two independent reviewers assessed and further screened the full-text articles of the potentially eligible studies. A third reviewer resolved any disagreements regarding the inclusion of studies at both stages. The outcomes of this screening process are presented in a PRISMA flow diagram.

### 2.5. Data Extraction

We extracted data using a systematically developed form in Microsoft Excel that included all the necessary fields to achieve the review’s objectives. The data extraction table template is available in Supplementary File S3.

The key indicators that we prioritized were infection-related data (type of infection, resistant pathogen, and intervention), economic evaluation-related information (type of economic evaluation, main outcome interest, and reported outcomes), and limitations/challenges reported in the studies. This review focused on six economic evaluation types, defined in the section ‘Definition of economic evaluations’ below. Two impartial reviewers meticulously extracted information on study attributes, economic assessments, and any limitations documented in the eligible research studies.

### 2.6. Definitions of Economic Evaluations

Cost-effectiveness analysis (CEA): CEA refers to a full economic evaluation that considers both the cost and effectiveness of each alternative intervention, and enables the combination of relevant outcome measures with costs, allowing alternatives to be ranked based on their effectiveness relative to resource utilization [12].

Cost-benefit analysis (CBA): CBA is a full economic evaluation that systematically compares the costs and benefits of an intervention to assess its economic profitability. In health economics, CBA compares the expected costs of healthcare interventions with their benefits to determine the most efficient use of resources. It is crucial for making well-informed decisions that enhance patient outcomes and optimize healthcare expenditures [13].

Cost-utility analysis (CUA): CUA is a full economic evaluation and that is considered an essential tool for evaluating and comparing the costs and effects of alternative interventions. CUA involves comparing the additional cost of a program from a specific perspective with the incremental health improvement expressed in terms of quality-adjusted life years (QALYs). CUA is a valuable tool for decision-makers aiming to maximize population health within budget constraints [14].

Cost minimization analysis (CMA): CMA is a full economic evaluation that evaluates and compares the costs of alternative interventions, helping to assess the value for money of an intervention. It is advantageous when both quantity (life years) and quality of life matter, as it measures health effects in terms of both by combining them into a single measure of health: QALYs. CMA is also a valuable tool for decision-makers aiming to maximize population health within a budget constraint [15].

Cost of illness (COI): COI is a partial economic evaluation that is commonly used to measure the economic burden of a specific disease or condition. It aims to identify and measure all costs associated with a particular disease, including direct, indirect, and intangible costs. Direct costs include medical costs, such as diagnostic tests, physician visits, medications, and nonmedical costs, including (but not limited to) travel expenses incurred when obtaining care. Indirect costs encompass productivity losses, such as work or leisure time missed due to the disease. Intangible costs are more challenging to quantify but may include factors such as reduced quality of life. The result of a cost of illness analysis is expressed in monetary terms, providing an estimate of the total economic burden of the disease on society. Decision-makers can use this information to understand the economic impact of a disease and prioritize interventions [16,17].

Disease burden studies: The burden of disease refers to the total and cumulative consequences of a specific disease or a range of harmful diseases within a community. These consequences encompass various aspects, including health impacts, social implications, and societal costs. The concept highlights the gap between an ideal scenario where everyone lives free of disease and disability and the current health status, thereby quantifying the overall burden. One widely used method to quantify the global disease burden is the measure of disability-adjusted life years (DALYs). These studies are crucial for understanding health priorities and informing public health interventions [18,19].

### 2.7. Quality Assessment

Considering the heterogeneity of the study designs of the selected studies, the Mixed-Methods Appraisal Tool (MMAT) was adapted for the quality appraisal of the eligible studies. The MMAT is a critical appraisal tool used in systematic reviews and meta-analyses that is designed to assess the methodological quality of included studies. It covers five study types: qualitative research, randomized and nonrandomized trials, quantitative descriptive studies, and mixed-methods studies [20]. The evaluation was performed by assigning a score of 1 if the criteria were fully met, 0.5 if the requirements were partially fulfilled, and a score of zero if the criteria were not met or if there was insufficient evidence to draw a conclusion. The sum of the scores for each study was then converted into percentages. A study with a score of more than 75.0% was classified as high quality, 50.0–74.0% as average quality, and less than 50.0% as poor quality. Two reviewers independently appraised the quality of each study, and a third reviewer was engaged when any disagreement occurred during the initial assessments.

### 2.8. Data Synthesis

An overview of the selected studies was presented, including the study citations, study year, country, income status according to the WB income classification, geographical distribution according to the WHO region classification, and type of economic evaluation. The primary outcome interests of this review, the available evidence and the study limitations of economic evaluations of AMR, were presented according to the type of economic evaluation.

## 3. Results

### 3.1. Search Results

**Figure 1:**
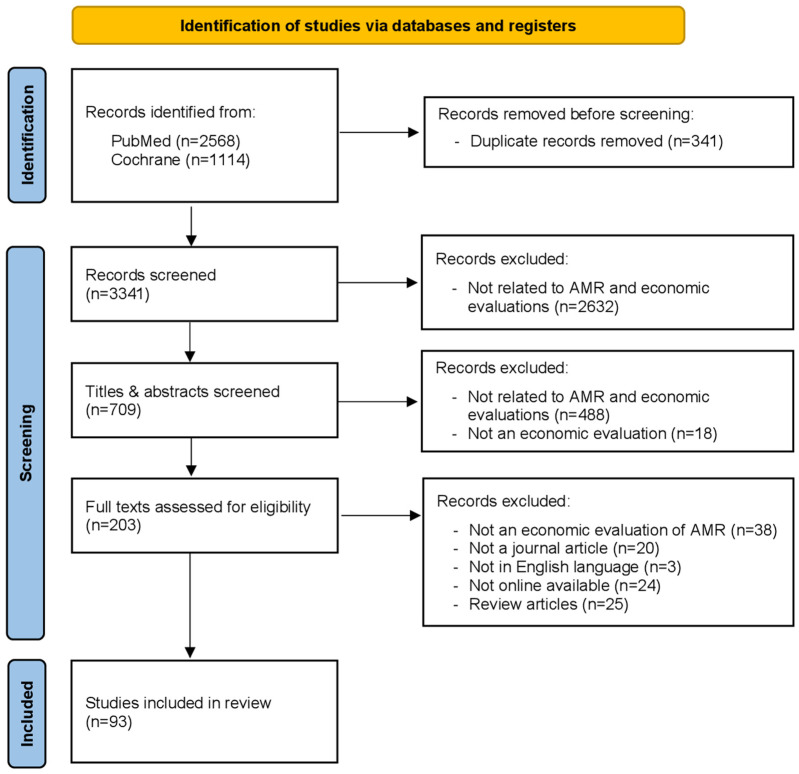
PRISMA flow diagram.

A total of 3682 records were retrieved during the record identification stage by searching the electronic databases PubMed (*n* = 2568) and Cochrane (*n* = 1114) (Table 1). After the title, abstract, and full-text screening, 93 articles met the criteria for inclusion in the review (Figure 1).

### 3.2. Quality Appraisal of Eligible Studies

Among the five study designs considered in the MMAT, this review included only three types of studies, including quantitative randomized controlled trials (*n* = 30, 32.3%), quantitative nonrandomized studies (*n* = 29, 31.2%), and quantitative descriptive studies (*n* = 34, 36.5%).

Among the quantitative randomized controlled trials, the majority (*n* = 24, 80.0%) were high-quality studies, five (16.7%) trials were of average quality, and one (3.3%) was rated as poor quality. However, among the quantitative nonrandomized studies, most of the studies (*n* = 15, 51.7%) were average-quality studies, followed by 12 (41.4%) high-quality studies and two (6.9%) poor-quality studies. Most of the quantitative descriptive studies were also high-quality studies (*n* = 21, 61.8%), followed by 10 (29.4%) average-quality studies and three (8.8%) poor-quality studies.

Overall, this review included 57 (61.3%) high-quality studies, 30 (32.3%) average-quality studies and six (6.5%) poor-quality studies (Supplementary File S4).

### 3.3. Overview of AMR Economic Evaluations

Among the selected studies (*n* = 93), 25 (26.2%) either did not report the type of economic evaluation or reported in a way that did not match our classification criteria. Therefore, we reclassified these into six types of economic evaluations (CEA, COI, CBA, CUA, CMA, or disease burden studies) based on their primary outcome of interest. The original and revised classifications are presented in Supplementary File S5.

Most studies were single-country analyses (*n* = 84, 90.3%) while 8 (8.6%) involved multiple countries, and one (1.1%) study did not specify the study location. Considering both single- and multiple country studies (*n* = 93), the total amounted to 162 distinct evaluations conducted across 58 countries.

**Figure 2:**
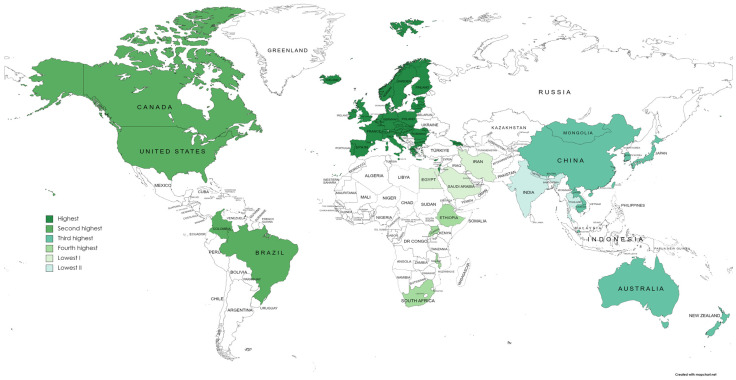
Countries reported AMR economic evaluations according to WHO region classification. Note: **Highest**: 85 analyses (52.5%) in 34 European countries (the UK, Netherlands, Germany, France, Greece, Belgium, Italy, Spain, Switzerland, Austria, Poland, Portugal, Slovakia, Slovenia, Bulgaria, Croatia, Cyprus, Czech Republic, Denmark, Estonia, Finland, Hungary, Iceland, Ireland, Israel, Latvia, Lithuania, Luxembourg, Malta, Norway, Romania, and Georgia, and Moldova); **second highest**: 35 analyses (21.6%) in four regions of the Americas (USA, Canada, Brazil, and Colombia); **third highest**: 29 analyses (17.9%) in 10 Western Pacific countries (Japan, Australia, Singapore, Republic of Korea, New Zealand, Taiwan, Cambodia, Lao PDR, Mongolia, and China); **fourth highest**: seven analyses (4.3%) in seven African countries (Malawi, Ethiopia, Uganda, and South Africa); **lowest I**: three analyses (1.9%) in three Eastern Mediterranean countries (Saudi Arabia, Egypt, and Iran); **lowest II**: three analyses (1.9%) in three South East Asian countries.

Considering the geographical distribution, Figure 2 presents the spread of AMR economic evaluations across regions based on the WHO regional classification. Across all 162 analyses from 93 studies in 58 countries, the European region accounted for the largest share (52.5%, 85 analysis across 34 countries), followed by the Americas (21.6%, 35 analysis across 4 countries), the Western Pacific region (17.9%, 29 analysis across 10 countries), the African region (4.3%, 7 analysis across 7 countries), the Eastern Mediterranean region (1.9%, 3 analysis across 3 countries), and the South-East Asia region (1.9%, 3 analysis across 3 countries) (Figure 2). Moreover, overview of the studies, including country and income classifications, is provided in Supplementary Files S6–S8.

Most of the studies were CEAs (*n* = 50, 53.7%), followed by COIs (*n* = 33, 35.4%), disease burden studies (*n* = 5, 5.4%), CBAs (*n* = 2, 2.2%), CUAs (*n* = 2, 2.2%), and a CMA (*n* = 1, 1.1%). The distribution of economic evaluations according to the WB income classification of countries is presented in Table 2.

### 3.4. Available Evidence of Economic Evaluations of AMR

All the studies were human-centered studies, and no animal- or environmental-related studies were found during this review. With respect to the study setting of all eligible studies, most studies were hospital-based (*n* = 74, 79.6%), including tertiary-care hospital-based studies (*n* = 9, 9.7%), followed by community-based (*n* = 8, 8.6%) and combined hospital- and community-based (*n* = 4, 4.3%) studies. Furthermore, seven studies (7.5%) did not reveal information related to the study setting. All the human center economic evaluations of AMR involved various infections, interventions, and resistant pathogens, which are presented according to the value type, unit of measurement and reported values in Appendix A.

### 3.5. Limitations Reported in Studies of AMR-Related Economic Evaluations

#### 3.5.1. Cost-Effectiveness Analysis

Among the 50 CEAs, the most common limitations reported were overestimation of results [21,22,23,24], underestimation of results [23,25,26,27,28,29,30,31], absence of primary data [21,28,32,33,34,35,36,37,38,39,40], lack of consideration of essential features or information [21,26,28,31,32,35,38,41,42,43,44,45,46,47,48,49,50], uncertainty [25,31,34,39,44,51,52,53], inadequate sampling approaches [21,22,29,35,41,44,54,55], assumption errors [23,30,32,47,55,56,57,58], errors in cost estimation [25,26,31,35,42,44,45,49,56,59,60,61,62,63,64], lack of generalizability/standardization of study results to the population [22,26,29,34,35,42,43,44,45,53,62], and other limitations [24,36,48,55,57,58,63]. More details are shown in Table 3. Three CEA studies out of 47 studies did not report limitations.

#### 3.5.2. Cost of Illness Studies

Among the 33 COI studies, the most common limitations reported were the underestimation of results [65,66], absence of primary data [66,67,68,69], no consideration of essential features or information [70,71,72,73,74], uncertainty [75,76], inadequate sampling approaches [6,66,73,77,78,79,80], assumption errors [75], errors in cost estimation [6,7,67,68,70,71,72,76,78,81,82,83,84,85,86], lack of generalizability/standardization of study results to the population [66,67,69,70,73,79,81,84,85,86,87,88], and other limitations [65,68,71,73,77,79,89], as shown in Table 4. However, study limitations were not reported in one COI study.

#### 3.5.3. Other Economic Evaluations: Disease Burden Studies, Cost-Benefit Analysis, Cost-Utility Analysis, and Cost Minimization Analysis

Among the disease burden studies, the absence of primary data [90,91,92,93] and the lack of consideration of essential features or data/information [90,93,94] were the significant limitations reported. The lack of generalizability/standardization of study results to the population was the most common limitation noted in the cost-benefit analysis [95,96], cost-utility analysis [97,98], and cost minimization analysis [99]. Further details are presented in Table 5.

### 3.6. Causes and Consequences of Study Limitations

**Figure 3:**
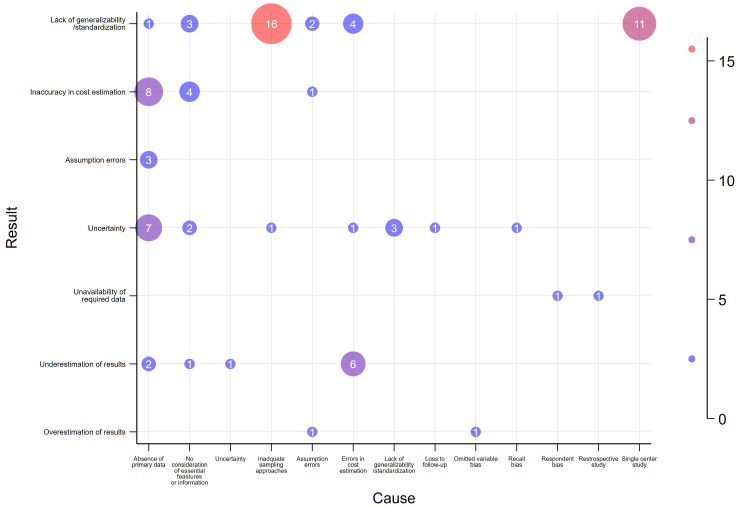
Causes and consequences of study limitations.

Figure 3 shows the interactions of limitations, showing how one limitation leads to another. The most common limitation interactions were the lack of generalizability/standardization of the study results to the population due to inadequate sampling approaches (reported in 16 studies) [6,21,29,35,41,42,44,54,55,66,67,77,78,79,92,99], single-center studies (reported in 11 studies) [22,41,44,55,73,78,79,80,84,96,98], errors in cost estimation (reported in 4 studies) [70,73,81,87], and no consideration of essential features or information [69,85,88] (reported in 3 studies). Additionally, the absence of primary data led to errors in cost estimation in 8 studies [35,40,45,47,52,60,67,85] and to the uncertainty of the study findings in 7 studies [21,25,31,34,39,52,53]. Furthermore, errors in cost estimation led to underestimations of results in 6 studies [25,27,28,29,31,66]. Further interactions are also presented in Figure 3.

## 4. Discussion

We conducted a systematic review of published literature up to July 2023, to synthesize all available evidence from economic evaluations related to AMR. Among the 3682 records identified, 93 eligible articles were included in our review. These articles included 50 CEAs, 33 COIs, five disease burden studies, two CBAs, two CUAs, and one CMA.

### 4.1. Available Evidence and the Evidence Gap in AMR Economic Evaluations

Nearly 75% of the studies originated from HICs, highlighting the disparity in evidence across varying income contexts. The absence of sufficient evidence in LMICs is particularly concerning, given that patients in these regions may require more potent medications, prolonged hospital stays, and additional medical interventions to treat AMR-related diseases. In resource-constrained settings, managing antibiotic-resistant infections poses greater challenges and incurs higher costs [100]. Conducting economic evaluations related to AMR can aid in allocating resources effectively in such resource-limited contexts. The significance of evidence in LMICs becomes apparent when considering the substantial 76% increase in antibiotic usage between 2000 and 2018, while HICs maintained relatively stable consumption during the same period [101]. Furthermore, in contrast to HICs, LMICs and LICs exhibit higher rates of antibiotic misuse due to overuse and self-prescription [102]. Given these challenges, strengthening the global evidence concerning AMR in LMICs is imperative.

Studies are more abundant in the region of Americas, the Western Pacific and European regions. However, only 2–3 articles were published for each African, Southeast Asian, and Eastern Mediterranean region. This pattern suggests that while numerous studies originate from HICs, they predominantly focus on the Americas, Western Pacific, and European regions rather than HICs in other regions. Nevertheless, expanding the evidence base for economic evaluations in other regions is crucial. A recent systematic review revealed that countries in the African region reported 18% higher rates of antibiotic misuse than other nations did [102]. Additionally, the World Health Organization has highlighted the substantial challenges faced by the African region due to inadequate enforcement and rapid proliferation of antibiotic-resistant strains resulting from misuse and overuse of antibiotics. These issues pose a significant threat to Africa’s public health and healthcare system, leading to increased morbidity, mortality, and healthcare expenses [103,104,105,106]. Furthermore, the Southeast Asia region faces significant challenges, as it is at risk of the emergence and spread of AMR in humans. Factors contributing to this risk include poverty, inadequate sanitation, and the overuse of antibiotics [107]. Moreover, a study conducted across 139 hospitals in seven Eastern Mediterranean countries revealed high overall hospital antimicrobial usage [108]. Considering the above findings, strengthening the global evidence concerning AMR in other regions where evidence is lacking is imperative.

Furthermore, despite the high importance and necessity of the One Health Approach for understanding and mitigating AMR, all the available economic evaluations have focused on human health. No studies were found in the Animal or Environmental related fields. This represents a limitation of the one health approach, which aims to sustainably balance the health of people, animals, and ecosystems [109]. A recent systematic review emphasized the importance of one health approach in AMR research, particularly for informing AMR policy decisions [110]. However, achieving these goals remains uncertain due to the lack of evidence related to animal and environmental health.

Additionally, community-based health studies play a crucial role in contextual learning within real-world settings, allowing us to understand health issues, observe health behaviors, and explore social determinants [111]. Unfortunately, the review revealed that most of the studies were conducted in hospital settings, leaving a gap in evidence for community-based evidence.

### 4.2. Limitations Reported in AMR Economic Evaluations and Their Interactions

We identified nine significant limitations associated with AMR economic evaluations: overestimation of results, underestimation of results, absence of primary data, no consideration of essential features or information, uncertainty, inadequate sampling approaches, assumption errors, errors in cost estimation, lack of generalizability/standardization of study results to the population, etc. When reviewing the limitations of AMR economic evaluations, we found that one limitation often leads to another, creating interconnected links among these limitations. The most common interaction occurs with the lack of generalizability/standardization of study results to the population. This limitation is influenced by other limitations, such as inadequate sampling approaches, reliance on single-center studies, errors in cost estimation, and no consideration of essential features or information.

Inadequate sampling approaches occur because a sample may not exhibit precisely the same behavior as the larger population from which it was selected. It may either underrepresent or overrepresent the study population, especially due to the non-random nature of the sample. For these reasons, inadequate sampling approaches are often recognized as one of the major reasons for the uncertainty and limited generalizability of the study findings [112,113]. On the other hand, single-center studies (a type of sampling error), which are conducted in a single hospital, clinic, or research institution, often involve a homogeneous sample due to limited diversity, as all data collection, participant recruitment, and intervention occur within this location. Therefore, the findings of these studies are generated under context-specific factors and geographical bias, which ultimately affect their generalizability [114,115].

Moreover, we identified the presence of a lack of applicability, and the uncertainty of the study results due to errors in cost estimations [25,27,28,29,31,66]. Errors in the data analysis could affect the applicability of the study findings, as inaccurate measurements or misclassification undermine the validity of the study results. According to this review, one of the reasons for errors in cost estimations is the absence of primary data required for the analysis, which is an uncontrollable limitation, as evidenced by 14 studies [21,25,31,34,35,39,40,45,47,52,53,60,67,85]. Furthermore, the lack of consideration of essential data/information, which could be due to a lack of data/information, also affects the generalizability and standardization of the study results to the population [69,85,88].

Consequently, considering the potential drawbacks and limitations, several critical factors should receive greater attention while addressing existing gaps in evidence. These factors include sample characteristics (such as the representativeness of the sample), the study context (for example, whether it occurs in a controlled laboratory environment), the relevance of the study over time (given societal, technological, and environmental changes), measurement tools, research design, and cultural and geographical considerations, which could impact the generalizability or standardization of study outcomes [113,116,117].

## 5. Strengths and Limitations

The key advantage of this review lies in our utilization of a comprehensive and sensitive search strategy to capture all published articles related to economic evaluations of AMR. Additionally, we deliberately avoided restricting the search time frame, ensuring that all relevant publications, regardless of their publication date, were included in our analysis up to July 2023.

However, several limitations are associated with this review. First, our search was confined to two research databases. Second, we did not consult the reference lists of eligible studies. Third, we focused exclusively on peer-reviewed journal articles, excluding gray literature, which might have restricted the inclusion of relevant information from published or unpublished sources. Fourth, our search was limited to English-language content, potentially introducing language bias. Fifth, we excluded articles that were not available online and did not contact corresponding authors for access to relevant publications. Finally, we categorized other types of economic evaluations into six predefined categories, which could introduce misclassification bias.

## 6. Conclusions and Recommendations

This review sheds light on the existing evidence gaps in LMICs, with a particular focus on the African, Southeast Asian, and Eastern Mediterranean regions. These gaps extend to the community-based approach, whereas the One Health Approach suffers from a complete lack of studies related to animal and environmental aspects. Additionally, the review identifies a chain of interrelated limitations, where one limitation often leads to another. The most common limitation lies in the lack of generalizability or standardization of the study outcomes to the population.

It is imperative that future studies address the identified evidence gaps more effectively. Specifically, researchers must focus on tackling the root causes of the most common limitations. These limitations, if left unaddressed, could significantly impact the validity of the study findings. Moreover, addressing these gaps would contribute to evidence-informed policy development aimed at combating AMR.

## Figures and Tables

**Table 1:** Database-specific search strategy and number of results per database.

Database	Search Strategy	Number of Records
PubMed	(“antimicrobial*”[Title/Abstract] OR “antibiotic*”[Title/Abstract] OR “antibacterial*”[Title/Abstract] OR “antiviral*”[Title/Abstract] OR “antifungal*”[Title/Abstract] OR “antiparasitic*”[Title/Abstract]) AND (“resistan*”[Title/Abstract] OR “overus*”[Title/Abstract] OR “misus*”[Title/Abstract]) AND (“economic evaluation”[Title/Abstract] OR “economic burden”[Title/Abstract] OR “cost effective*”[Title/Abstract] OR “cost utility”[Title/Abstract] OR “cost benefit”[Title/Abstract] OR “cost of illness”[Title/Abstract] OR “cost minimization”[Title/Abstract] OR “incremental cost-effectiveness ratio”[Title/Abstract] OR “qaly*”[Title/Abstract] OR “quality adjusted life year*”[Title/Abstract] OR “daly*”[Title/Abstract] OR “disability adjusted life year*”[Title/Abstract])	2568
Cochrane	((Antimicrobial*):ti,ab,kw OR (Antibiotic*):ti,ab,kw OR (Antibacterial*):ti,ab,kw OR (Drug*):ti,ab,kw OR (Medicine*):ti,ab,kw OR (Antiviral*):ti,ab,kw OR (Antifungal*):ti,ab,kw OR (Antiparasitic*):ti,ab,kw) AND ((Resistan*):ti,ab,kw OR (Overus*):ti,ab,kw OR ((Over NEXT usage*)):ti,ab,kw OR (Misus*):ti,ab,kw) AND ((“Economic evaluation”):ti,ab,kw OR (“Economic burden”):ti,ab,kw OR ((Cost NEXT effective*)):ti,ab,kw OR (“Cost utility”):ti,ab,kw) AND ((“Cost benefit”):ti,ab,kw OR (“Cost of illness”):ti,ab,kw OR (“Cost minimization”):ti,ab,kw OR (“Incremental cost-effectiveness ratio”):ti,ab,kw OR (QALY*):ti,ab,kw AND ((Quality adjusted life NEXT year*)):ti,ab,kw OR (DALY*):ti,ab,kw OR ((Disability adjusted life NEXT year*)):ti,ab,kw)	1114
Total records		3682

**Table 2:** AMR economic evaluations according to the WB income classification.

Income Level	CEA		COI		Other Economic Evaluations **		Total *	
	*n*	%	*n*	%	*n*	%	*n*	%
High-income countries	39	60.0	22	33.8	4	6.2	65	100
Upper middle-income countries	8	57.1	5	35.7	1	7.2	14	100
Lower middle-income countries	0	0	3	100	0	0	3	100
Low-income countries	1	50.0	1	50.0	0	0	2	100
Total *	48	57.1	31	36.9	5	6.0	84	100

Note: * Only single country studies were included in this classification. ** CBA, CUA, and CMA studies were included in other economic evaluations.

**Table 3:** Limitations associated with AMR-related CEA.

Reported Limitations	Description/Cause(s) of the Reported Limitation
Overestimation of results	Patients initially treated with ertapenem (treatment I) are less likely to show success with imipenem (treatment II) because it is in the same class as ertapenem. As a result, the QALYs gained with ertapenem were arguably overestimated [21]. Accurately estimating the incremental length of hospital stay attributable to *S.aureus* bacteremia is affected by omitted variables and simultaneity biases. Therefore, the estimations might be relatively high [22]. The effectiveness of genotypic antiretroviral resistance testing may have been overestimated if failure to prescribe optimal therapy [23]. Overestimation of total cost due to the unrealistic assumption; all antibiotic treatment days are associated with nosocomial pneumonia [24].
Underestimation of results	Only included production loss for working-aged people (20–64 years). This probably led to an underestimation of the true cost-effectiveness [25]. The model used does not capture the difference in resistance profile between two antibiotic groups, which resulted in an underestimation of the cost and QALY advantages observed over time [30]. The effectiveness of genotypic antiretroviral resistance testing may have been underestimated if, over time, clinicians learn to improve their choice of GART-guided therapies [23]. The difference in benefits between the two gonorrhea treatment strategies may have been underestimated [26]. Hospital length of stay was likely underestimated as healthcare resource utilization and cost data in the clinical trial were only measured through the end of the study visit for all modified intent to treat patients [27]. Underestimating incremental costs as suspected cases might be included in the cohort [28]. Underestimation of length of stay as measured healthcare resource use and costs through the end of study visits [29]. Underestimation of hospitalization costs as they were not adjusted for inflation over the years [31].
Absence of primary data	Only diabetic-specific survival estimates were used for patients with sequelae [21]. The study had to rely on the UK cost-effectiveness threshold as the unavailability of the European-wide threshold [32]. The published data on the utility of the various health states examined in the model is unavailable [33]. Lack of information on long-term survival and optimal duration of nivolumab [34]. The model utilized published data from other populations due to the unavailability of data on the long-term mortality and the health utility of patients with carbapenem-resistant *Klebsiella pneumoniae* bloodstream infection [35]. The absence of a specific ICD-9-CM diagnosis code for Nosocomial Pneumonia and our reliance on an algorithm to exclude patients thought to have community-acquired pneumonia [36]. Due to the unavailability of used data on the prevalence of NS5A resistance from the European and USA population, they may not reflect the prevalence in the UK population [37]. Lack of reported data on the relationship between resistance and clinical failure/the model requires using population-specific efficacy data, and the meta-analyses found in the literature were unsuitable [38]. High amount of missing data [39]. Unable to directly assess esketamine’s cost-effectiveness versus alternatives due to the absence of comparative outcomes data [40]. Data sources for this study were unavailable daily, which could lead to time-dependent bias [28].
No consideration of essential features or information	Did not subgroup chronic gastritis patients into nonatrophy, atrophy, and intestinal metaplasia groups, which are other potential factors that might affect *H. pylori* eradication [41]. Only diabetic-specific survival estimates were considered for patients with sequelae/adverse events [21]. The intervention may not be compared with the most relevant alternatives as this is a placebo-controlled trial [32]. The decision analytic model focused on mild to moderate Community-acquired pneumonia (CAP) but did not consider severe CAP [42]. Did not consider gonorrhea-positive patients coinfected with another organism, which would affect patient pathways and treatment options [43]. The Markov model did not consider the relapse rates and rehospitalizations [35]. A budget impact analysis was not performed [44,45]. The model excludes several significant factors, including the potential benefits to the PTSD patients’ families, the broad reduced risks, domestic violence, severity of substance abuse and the criminal justice system’s involvement [46]. The value of the intervention in which patients shared rooms has not been explored [47]. Treatment-related adverse effects were not addressed [38]. Did not account for different anatomic locations of gonorrhea infections and the import of gonorrhea infections with resistant strains [26]. Did not evaluate the cost-effectiveness of testing for other resistance variants (e.g., NS5B and NS3) and the performance characteristics (e.g., sensitivity, specificity) of a diagnostic test [45]. Suspected cases and other hospital-acquired and multidrug-resistant infections were not actively excluded from the cohort [28]. Did not specifically compare the periods before or after the diagnosis of infection [48]. The analysis of the feasibility of obtaining the AMR reductions has not been validated [49]. The analysis did not capture the broader effects of hearing loss on the ability to work [50]. Possible differences in future populations, demographic changes (including the growth in the number of older people)/The constraints of the model led to the use of a limited number of pathogens (e.g., three pathogens in this study)/The diseases and pathogens analyzed were limited [31].
Uncertainty	Uncertainty of input data. Uncertainty of input data due to sampling errors and lack of data [21]. Not all the participants completed all the parts of the questionnaire/recall bias as collecting data over six months from the patients with depression [39]. Data were generated not from the fundamental analyses but from the established two-parameter Weibull survival model [51]. The available data was that survival time was censored on the date of progression for patients who had not died [52]. The vaccine protection duration is not fully understood and is uncertain [53]. Uncertainty of the results. Uncertainty about the results as input values and transmission probabilities were collected from different studies and combined in this study [25]. Uncertainty of the results due to mis-estimation influence of used data obtained from other sources [34]. The impact from a societal perspective is unclear due to the perspective taken in the analysis. The accuracy of cost-effectiveness may be restricted due to using the EQ-5D-3 L to assess the health-related utility [44]. The absence of empirical research led to reliance on expert opinion, which made the uncertainty of the estimates of hospital length of stay [31].
Inadequate sampling approaches	Errors in the sampling method [21]. Small sample size—sample of 180 [41], sample of 263 [54], sample of 3000 [35], sample of 96 [55], sample of 1225 [29]. Single-center study [22,41,44,55].
Assumptions errors	Using false/unrealistic assumptions. Assuming that the cost of resistance is the same across all antibiotics regardless of antibiotic class and the sector of care/assuming that there is a linear relationship between prescribing and resistance; however, in practice, the impact will be lagged, and there may be a nonlinear relationship [32]. Assuming patients received all required/necessary treatments; however, patients may not receive all for various reasons, including disease progression and personal choice [56]. Antibiotics used to treat other Gram-positive and Gram-negative pathogens were assumed to be similar between both groups [55]. Assumed a general medicine ward set up with 20 single bedrooms, which may not be the configuration of all general medicine wards [47]. The assumption is that patients in the treatment completion state have the same health characteristics as the general population [57]. The use of resources in the clinical trial may not represent the actual clinical practice since clinical trials are conducted in a selected population that meets the inclusion and exclusion criteria [58]. Using simplification compared to routine practice: all patients are treated with piperacillin/tazobactam, or all patients are treated with ertapenem [30]. Using unproven assumptions about the mechanism of disease progression [23]. Using a higher number of assumptions [57].
Errors in cost estimation	Direct cost. limited to the cost of pharmaceutical treatments and the administration of those treatments, not taking into consideration the cost of treating other sequelae of castration-resistant prostate cancer, such as pain, spinal cord compression, or palliative care [56]. Only productivity loss was included, direct cost was missing [25]. Costs associated with prolonged hospitalization due to nephrotoxicity were ignored [35]. Direct nonmedical cost was not included [26,59]. The cost of monitoring vancomycin plasma levels is not included, which is necessary to obtain optimal results with this treatment/treatment costs of adverse reaction outcomes for both products, which could influence the global decision-making process [60]. The cost of treatment-related adverse events was not accounted for in the analysis [45]. Hospitalization costs have not been adjusted for inflation over the ten years [31]. Indirect cost. Did not consider indirect costs [35,59,61,62]. Costs of productivity loss were not considered [42,63,64]. The cost of welfare services was not included [44]. Not considering the additional costs associated with human and health resources of implementing approaches/future health costs associated with improved survival are omitted [49].
Lack of generalizability/standardization of study results to the population	Study results cannot be extrapolated to the patients with severe community-acquired pneumonia, only for the moderate patient population [42]. Lack of generalizability and applicability due to AMR rates constantly changing/generalizable only to England, not other countries [43]. The true cost-effectiveness could differ from the study findings as real-life survival or nivolumab use varies substantially from the values in this study [34]. Findings cannot be generalized for other Chinese provinces and countries due to significant variations in healthcare resources and epidemiology of *K. pneumoniae* resistance [35]. Cannot be generalizable to other populations as the participants may not represent the typical background of depressed patients in Japan, only for hospitalized patients [44]. Cannot be fully generalizable to hospitals with different care levels or settings in other countries [22]. Study results were restricted to men and the health benefits in men; only the population of men who had sex with men [26]. Generalizability of the results for other races/ethnicities or in other countries may be limited due to the heterogeneity of payer perspectives and the country-specific epidemiologic data used [45]. It is generalizable to other settings where only introducing drug-resistant strains may lead to prolonged outbreaks of typhoid fever [53]. Generalizable only to the population of the study setting, and the applicability of this data to other patients is also limited [62]. The vancomycin dosing used in the study may not reflect real-world dosing practice patterns [29].
Others	Lost to follow-up [57]. Retrospective study [48,55,58,63]. Using proxy estimates of costs due to the unavailability of claims data [36]. Respondent bias as using the Delphi method [24].

**Table 4:** Limitations associated with AMR-related COI.

Reported Limitations	Description/Cause(s) of the Reported Limitation
Underestimation of results	The results were underestimated due to difficulties distinguishing between infection and colonization [65]. The results were underestimated due to underestimating severe pneumonia patients and medical costs [66]. The results were underestimated due to the changing behavior of the clinicians to fit the expected results in the observational study—Hawthorne effect [79].
Absence of primary data	Lack of availability of usable estimates of the bed day cost [67]. No related prospective trials were available for the similar patient population [68]. More data was missing from the study participants [69]. The database did not report laboratory-based information including microscopy on the infecting pathogens [66].
No consideration of essential features or information	Not possible to estimate the impact of adverse events onward costs [70]. Cannot distinguish colonization or infection, which incur significantly different costs due to the nature of the retrospective study [71]. Social burden due to deterioration of quality of life from illnesses was not considered/In the process of selecting matching patients for the control groups, some patients were not selected for the control groups and were excluded from the analyses [72]. Not including several essential patient subgroups [73]. Not including antibiotic exposure data [74].
Uncertainty	Uncertainty of the input data. Direct and indirect costs for this study were derived from the published literature and may not fully represent real-world costs [75]. Data sources depend on the coding quality of hospital records and do not reflect the actual costs to the hospital [76]. Uncertainty of the study results. Results were uncertain as the analysis is based on the population included in Phase III clinical trials and may not represent the overall TRD population/all patients were required to take their medication under medical supervision, which does not reflect real-world practice [75].
Inadequate sampling approaches	Small sample size—sample of 34 [77], sample of 99 [6], sample of 1539 [78]. Limited to the sample of one-region/single-center study [73,78,79,80]. Selection bias in MRSA cases was identified using anti-MRSA drugs [66].
Assumptions errors	Total employment (100%) is assumed when estimating the indirect cost [75]. All patients were assumed to follow a similar and consistent pattern of initiating a new line of therapy following a relapse, which may not represent the actual treatment among these patients [75].
Errors in cost estimation	Direct costs. Cost estimates may not reflect costs incurred at specific institutions [70]. The cost of multiple infections or pathogens was excluded [76]. Not considered all direct medical costs—only considered the excess cost to treat bloodstream infection [67], only considered the laboratory investigation cost [81]. Direct nonmedical costs were excluded [71,82]. Additional costs, such as investigations, follow-up outpatient visits, and management costs, were excluded [6,78,83]. Hospitalization costs were not separated into pre- and postdiagnosis of the infection [84]. Costs associated with medical equipment, staff time, and other additional aspects of treating a patient in the hospital were omitted [7]. Data were extracted from an administrative claims database; therefore, the findings are subject to potential miscoding [85]. Indirect costs. Not all the indirect costs are accurately estimated/included [81]. Excluded indirect costs [68,71,72,78,82,86].
Lack of generalizability/standardization of study results to the population	Lack of generalizability as the cost values were not representative of the whole region [70,81,87]. Results were based on acute care hospital data, which may not apply to other healthcare settings [67]. Lack of generalizability to other regions as the study took place only in South Texas [79]. The generalizability issue was due to the experience of trial participants, which was different from that of patients seen in routine practice [69]. The model built under which the clinical trial was performed may differ from real-life clinical practice in Germany [86]. The implications are only valid for hospital-based, as the impact of AMR in the community has not been included [88]. Lack of generalizability due to the samples may not represent all severe community-onset pneumonia patients [66]. The study involved only two institutions, and the findings may not be generalizable to other institutions [84]. The costs used to estimate lost productivity from hospitalization and death were national averages and may not apply to a sicker population/Costs and mortality rates were measured in a subset of hospital patients who were at high risk and suffering with severity of illness; therefore, results cannot be applied to all patients in the community [73]. The treatment failure algorithm used to identify patients with treatment-resistant depression (TRD) may not be representative of all patients with TRD [85].
Others	Retrospective study [65,68,71,73,79,89]. Methodological errors. Healthcare facilities were not randomly selected [77]. Patients were not blinded [79].

**Table 5:** Limitations associated with other economic evaluations of AMR.

Type of Economic Evaluation	Reported Limitations	Description/Cause(s) of the Reported Limitation
Disease burden (*n* = 5)	Absence of primary data	The unavailability of national surveillance data in Greece compelled the reliance entirely on data from the only existing national point prevalence survey data [90]. Many of the parameters required for estimating DALYs were borrowed from previous studies/data about the length of stay in Japanese hospitals, which were scarce [91]. In the absence of Australian estimates of morbidity and mortality attributable to AMR, used estimates from Queensland to project to the Australian population [92]. Published data on clinical responses to treatments is considered lacking [93].
	No consideration of essential features or information	The strength of the evidence supporting each parameter estimate was not graded based on the clinical outcome studies’ statistical analysis method/Did not adjust the models for age-specific risks, coinfections, appropriateness of antibiotic therapy, or type of care [94]. The analysis did not account for treatment factors [90]. Drug allergies or adverse events were not considered in the model [93].
	Others	Underestimation of results—Underestimation due to the exclusion of patients due to missing data [91]. Uncertainty of input data—The input data were uncertain as the data for the disease models were retrieved from systematic literature reviews, which were varied in the representativeness of required evidence, availability and quality [94]. Assumption errors-Assumed there are common transition probabilities for all subgroups [94]. Errors in cost estimates—did not consider societal cost, which covers a range of health services, health infrastructure, and disease prevention [92]. Lack of generalizability/applicability of study results to the population—Used data limited in representing the entire Australian population [92].
CBA (*n* = 2)	Lack of generalizability/standardization of study results to the population	Lack of generalizability to other settings [95]. Data on unit costs were mainly derived from the university hospital and thus may not be comparable to those at other hospitals [96].
	Others	Absence of primary data—unavailability of molecular typing necessary for the analysis [95]. Assumption errors—assumption of no differences in patient contacts or care due to the use of gowns [95]. Errors in cost estimation—Omission of blood culture costs [95].
Cost–utility analysis (*n* = 2)	Lack of generalizability/standardization of study results to the population	Applicable only to a patient population with similar characteristics to those included in this study [97]. The information may not apply to patients with newly developed healthcare-associated urinary tract infections, and the generalization of these results in different clinical settings with less severe patients should be limited [98].
	Others	Absence of primary data—The long-term quality of life and survival were extrapolated from the literature [97]. No consideration of essential features or information—Did not account for several factors, such as the emergence of drug resistance during treatment, the possibility of antibiotic-related adverse reactions, and the risk of other healthcare-associated infections during hospitalization [98]. Single-center study [98].
CMA (*n* = 1)		Inadequate sampling approaches—lower sample size (*n* = 50) [99]. Lack of generalizability/standardization of study results to the population—results did not apply to other healthcare systems [99].

## Data Availability

All the data are included in the manuscript and in the public domain.

## Supplementary Materials

The following supporting information can be downloaded at: https://www.mdpi.com/article/10.3390/antibiotics14111072/s1, Supplementary File S1: PRISMA 2020 for Abstracts Checklist [118]; Supplementary File S2: PRISMA 2020 Checklist [118]; Supplementary File S3: Data extraction template; Supplementary File S4: Quality appraisal of eligible studies; Supplementary File S5: Revised types of economic evaluations; Supplementary File S6: AMR Economic evaluation by income classification; Supplementary File S7: AMR Economic evaluation by countries; Supplementary File S8: Characteristics of selected studies.

## Abbreviations

AMR	Antimicrobial resistance
CBA	Cost–benefit analysis
CEA	Cost-effectiveness analysis
CMA	Cost minimization analysis
COI	Cost of illness
CUA	Cost–utility analysis
DALYs	Disability-Adjusted Life Years
DHSC	Department of Health and Social Care
HICs	High-income countries
Lao PDR	Lao People’s Democratic Republic
LICs	Low-income countries
LMICs	Lower- and middle-income countries
MeSH	Medical subject headings
MMAT	Mixed-methods appraisal tool
PRISMA	Preferred Reporting Items for Systematic Reviews and Meta-Analyses
QALYs	Quality-adjusted life years
UK	United Kingdom
USA	United States of America
WB	World Bank
WHO	World Health Organization

## Appendix A

**Table A1:** Available evidence from economic evaluations of AMR.

Type of Economic Evaluation	Citation (Study Conducted Year)	Infection/Ill Health Condition	Resistant Pathogen	Intervention/Treatment (Type of Antibiotic/Drug)	Sector/Setting	Value Type	Unit of Measurement	Value
CEA	Jansen et al. 2009 [21] (2006)	Diabetic foot infection	1. *Enterobacteriaceae* 2. Methicillin-resistant *Staphylococcus aureus* 3. *Enterococci* 4. *Pseudomonas aeruginosa*	1. Ertapenem (Treatment I) 2. Piperacillin/tazobactam (Treatment II)	Human/Setting—no data	Lifetime QALYs	Years	Treatment I and II: 12.13 and 10.97
						Direct medical cost (Drug) per patient	Great British Pound (GBP)	Treatment I and II: 456.00 and 781.00
						Total cost	GBP	Treatment I and II: 5072.00 and 8537.00
						QALY savings	Years	Treatment I vs. II: 1.16
						Cost per QALY saved	GBP	Treatment I vs. II: 407.00
	Martin et al. 2008 [42] (2006)	Community-acquired pneumonia	1. *S. pneumoniae* 2. *H. influenzae* 3. Atypical pathogens (*Mycoplasma pneumoniae*, *Chlamydia pneumoniae*, *Legionella* spp.)	1. Moxifloxacin/Coamoxiclav 2. Coamoxiclav/Clarithromycin 3. Cefuroxime/Moxifloxacin 4. Clarithromycin/Moxifloxacin	Human/Hospital and community-based	First-line treatment failure	Percentage	Treatment I to IV: 5, 16, 19, 18
						Second-line treatment failure	Percentage	Treatment I to IV: 4, 13, 16, 15
						Hospitalization rate	Percentage	Treatment I to IV: 1, 4, 4, 4
						Death rate	Percentage	Treatment I to IV: 0.01, 0.04, 0.03, 0.03
						Direct healthcare costs per episode	Euro	Treatment I to IV: 143.53, 221.97, 211.16, 192.79
	Harding-Esch et al. 2020 [43] (2015–2016)	*Neisseria gonorrheae*	No data	Standard care vs. Antimicrobial resistance point-of-care testing (AMR POCT); 1. A: Single POCT for ciprofloxacin; dual therapy 2. B: Dual POCT for azithromycin and ciprofloxacin; dual therapy 3. C: Dual POCT for ciprofloxacin and azithromycin; dual therapy 4. D: Single POCT for azithromycin; monotherapy 5. E: Single POCT for ciprofloxacin; monotherapy	Human/Hospital-based	Total additional cost	GBP	1,098,386.00/1,237,676.00/1,210,330.00/415,516.00/601,414.00 ^a^
						Additional cost per patient	GBP	28.26/31.84/31.14/10.69/15.47 ^a^
						Number of optimal treatments gained	Number of patients	895/1660/1449/1002/895 ^a^
						Additional cost per optimal treatment gained	GBP	1226.97/745.44/835.39/414.67/671.82 ^a^
	Wysham et al. 2017 [33] (2015)	Ovarian cancer	Platinum-resistant recurrent ovarian cancer	Adding bevacizumab to single-agent chemotherapy	Human/Hospital-based	ICER associated with B + CT per QALY gained	United States Dollar (USD)	410,455.00
						ICER associated with B + CT per PF-LYS	USD	217,080.00
						Cost per person (CT)	USD	57,872.00
						Cost per person (B + CT)	USD	117,568.00
						Incremental cost (B + CT)	USD	59,696.00
	Morgans et al. 2022 [62] (2020)	Prostate cancer	Metastatic castration-resistant prostate cancer previously treated with docetaxel and an ARTA	Cabazitaxel (Treatment I) vs. a second androgen receptor-targeted agent (Treatment II)	Human/Hospital-based	Cost of symptomatic skeletal events	USD	Treatment I and II: 498,909.00 and 627,569.00
						Cost of adverse events	USD	Treatment I and II: 276,198.00 and 251,124.00
						Cost of end-of-life care	USD	Treatment I and II: 808,785.00 and 1,028,294.00
						Hospitalization cost	USD	Treatment I and II: 1,442,870.00 and 1,728,394.00
						Length of hospital stay avoidable	Days	Treatment I vs. II: 58
						Length of ICU stay avoidable	Days	Treatment I vs. II: 2
						Total Cost saving	USD	Treatment I vs. II: 323,095.00
						Length of hospital stay	Days	Treatment I vs. II: 260 and 318
						Length of ICU stay	Days	Treatment I vs. II: 6 and 8
	Oppong et al. 2016 [32] (2012)	Lower respiratory tract infections	No data	Amoxicillin	Human/Setting—No data	Estimated threshold for the cost of resistance	GBP	4.98 for 20,000.00 per QALY threshold, and 8.68 for 30,000.00 per QALY
						Difference in cost between amoxicillin and placebo groups with the inclusion of possible values for the cost of resistance	GBP	With US data = 66.09
								With European data = 4.42
								With Global data = 218.25
						Incremental cost-effectiveness ratios (per QALY gained with the US, European, and global data, respectively)	GBP	With US data = 178,618.00
								With European data = 11,949.00
								With Global data = 589,856.00
	Yi et al. 2019 [41] (No data for the study conducted year)	*Helicobacter pylori* infection	No data	1. Furazolidone-based quadruple therapy (FZD) 2. Clarithromycin-based quadruple therapy (CLA)	Human/Hospital-based	Total drug cost (per single treatment course)	USD	FZD = 70.05
								CLA = 89.43
						Effectiveness	Percentage	FZD = 93.26
								CLA = 87.91
						Cost-effectiveness ratio	Ratio	FZD = 0.75
								CLA = 1.02
						Incremental cost-effectiveness ratio	Ratio	−3.62
	Kirwin et al. 2019 [28] (2011–2015)	No data	Methicillin-resistant *Staphylococcus aureus*	No data	Human/Hospital and community-based	Incremental inpatient costs associated with hospital-acquired cases	Canadian dollars (CAD)	For colonization: 31,686.00 (95% CI: 14,169.00–60,158.00)
								For infection: 47,016.00 (95% CI: 23,125.00–86,332.00)
						Incremental inpatient costs associated with community-acquired cases	CAD	For colonization: 7397.00 (95% CI: 2924.00–13,180.00)
								For infection: 14,847.00 (95% CI: 8445.00–23,207.00)
						Incremental length of stay—hospital-acquired infections	Days	35.2 (95% CI: 16.3–69.5)
						Incremental length of stay—community-acquired infections	Days	3.0 (95% CI: 0.6–6.3)
	Evans et al. 2007 [48] (1996–2000)	No data	Gram-negative bacteria	1. Ciprofloxacin 2. Cefazolin 3. Vancomycin 4. Fluconazole 5. Metronidazole 6. Gentamicin 7. Piperacillin/tazobactam 8. Cefepime 9. Cefoxitin 10. Amphotericin 11. Cotrimoxazole 12. Clindamycin 13. Imipenem 14. Meropenem	Human/Hospital-based	Hospital costs (median)	USD	80,500.00 vs. 29,604.00 (*p* < 0.0001)
						Antibiotic costs (median)	USD	2607.00 vs. 758.00 (*p* < 0.0001)
						Hospital length of stay (median)	Days	29 vs. 13 days (*p* < 0.0001)
						Intensive care unit length of stay (median)	Days	13 days vs. 1 day (*p* < 0.0001)
						Estimated incremental increase in hospital cost [median (95% CI)]	USD	11,075.00 (3282.00–20,099.00)
	Pollard et al. 2017 [56] (2013)	Metastatic castration-resistant prostate cancer	No data	1. Sipuleucel-T (Sip-T) 2. Eenzalutamide (Enzal) 3. Abiraterone (Abir) 4. Docetaxel (Doc) 5. Radium-223 (Rad) 6. Cabazitaxel (Cabaz) 7. Prednisone (Pred) 8. Leuprolide (Leup) 9. Deno (Deno)	Human/Setting—no data	Unit Cost	USD	Sip − T = 31,000.00
								Enzal = 7450.00
								Abir = 5390.00
								Pred (10 mg daily) = 0.40
								Doc = 1515.62
								Rad = 11,500.00
								Cabaz = 7773.00
								Leup (3 mo depot) = 141.75
								Deno = 1587.30
						Total cost	USD	Sip − T = 98, 860.25
								Enzal = 61,835.00
								Abir = 43,216.00
								Doc = 16,235.26
								Rad = 73,700.51
								Cabaz = 50,038.79
								Leup(3 mo depot) = 2477.46
								Deno = 71,499.65
						Incremental cost	USD	Sip − T = 106,117.00
								Sip − T + Enzal = 68,384.00
								Sip − T + Enzal + Abir = 50,119.00
								SipT + Enzal + Abir + Doc = 245,103.00
								Sip − T + Enzal + Abir + Doc + Rad = 80,072.00
								Sip − T + Enzal + Abir + Doc + Rad + Cabaz = 54,287.00
						ICER	Ratio	Sip − T = 312,109.00
								Sip − T + Enzal = 220,594.00
								Sip − T + Enzal + Abir = 151,876.00
								SipT + Enzal + Abir + Doc = 207,714.00
								Sip − T + Enzal + Abir + Doc + Rad = 266,907.00
								Sip − T + Enzal + Abir + Doc + Rad + Cabaz = 271,435.00
	Larsson et al. 2022 [25] (2015–2019)	Febrile urinary tract infections	*Escherichia coli*	Temocillin vs. Cefotaxime	Human/Hospital-based	Treatment cost (Temocillin, Cefotaxime, Difference)	Euro	5,620,393.00, 443,258.00, 5,177,135.00
						Healthcare costs (Temocillin, Cefotaxime, Difference)	Euro	2,480,820.00, 4,531,007.00, −2,050,187.00
						Production loss costs (Temocillin, Cefotaxime, Difference)	Euro	190,572.00, 348,063.00, −157,491.00
						Total costs (Temocillin, Cefotaxime, Difference)	Euro	8,291,785.00, 5,322,328.00, 2,969,457.00
						QALY (Temocillin, Cefotaxime, Difference)	Years	36,653.00, 36,576.00, 77.16
						ICER	Ratio (Euro/QALY)	38,487.00
	Rao et al. 1988 [119] (1986–1987)	No data	Methicillin-resistant *Staphylococcus aureus*	No data	Human/Hospital-based	Cost of outbreak—total reimbursement to the hospital	USD	34,415.00
						Discrepancy between the cost of treatment and financial reimbursement	USD	79,905.00
						Cost of MRSA eradication	USD	9984.00
	Tu et al. 2021 [54] (2019)	1. Bone Metastases Breast cancer 2. Castration-resistant prostate cancer	No data	12 vs. 4-Weekly Bone-Targeted Agents (BTA)	Human/Hospital-based	Total cost of treatment	Canadian dollars	4-weekly BTA = 8965.03
								12-weekly BTA = 5671.28
								Incremental = −3293.75
						QALY	Years	4-weekly BTA = 0.605
								12-weekly BTA = 0.612
								Incremental = 0.008
						ICER (∆ cost/∆ QALY)	Descriptive	12-weekly dominates 4-weekly
						Incremental net benefit (INB)	Canadian dollars	3681.37
	Wang et al. 2015 [120] (2006–2014)	Chronic hepatitis B with adefovir dipivoxil resistance	No data	1. Lamivudine (LMV) 2. Telbivudine (LdT) 3. Entecavir (ETV)	Human/Hospital-based	Cost	USD	LMV + ADV = 3024.00, LdT + ADV = 3292.80, ETV + ADV = 5107.2
						Cost-effectiveness ratio of negative conversion of HBV DNA	Ratio	LMV + ADV = 33.3, LdT + ADV = 35.4, ETV + ADV = 544.0
						Incremental cost-effectiveness ratio of negative conversion of HBV DNA	Ratio	LdT + ADV = 134.4, ETV + ADV = 718.3
						Cost-effectiveness ratio of seroconversion of HBeAg/HBeAb	Ratio	LMV + ADV = 63.4, LdT + ADV = 67.5, ETV + ADV = 99.5
						Incremental cost-effectiveness ratio of seroconversion of HBeAg/HBeAb	Ratio	LdT + ADV = 244.4, ETV + ADV = 578.7
						Cost-effectiveness ratio of nongenotypic mutation	Ratio	LMV + ADV = 31.3, LdT + ADV = 33.7, ETV + ADV = 52.4
						Incremental cost-effectiveness ratio of nongenotypic mutation	Ratio	LdT + ADV = 268.8, ETV + ADV = 2314.7
	Tringale et al. 2018 [34] (2017)	1. Platinum-resistant recurrent 2. Metastatic squamous cell carcinoma	No data	Nivolumab vs. Standard therapy	Human/Setting—No data	Overall cost increased (with Nivolumab)	USD	117,800.00
						Overall cost increased (with standard therapy)	USD	178,800.00
						Increased effectiveness of QALYs (with Nivolumab)	Years	0.40
						Increased effectiveness of QALYs (with standard therapy)	Years	0.796
						ICER	Ratio	294,400.00/QALY
	Wolfson et al. 2015 [57] (2013)	Multidrug-resistant tuberculosis	No data	Bedaquiline plus background regimen (BR)	Human/Hospital and Community-based	Total Cost	GBP	Bedaquiline + BR = 2,170,394.00, BR only = 2,403,442.00, Incremental (Bedaquiline + BR vs. BR) = −233,048.00
						Per patient cost	GBP	Bedaquiline + BR = 106,487.00, BR only = 117,922.00, Incremental (Bedaquiline + BR vs. BR) = −11,434.00
						Total QALYs gained	Years	Bedaquiline + BR = 105.09, BR only = 81.80, Incremental (Bedaquiline + BR vs. BR) = 23.28
						Per patient QALYs	Years	Bedaquiline + BR = 5.16, BR only = 4.01, Incremental (Bedaquiline + BR vs. BR) = 1.14
						Total DALYs lost	Years	Bedaquiline + BR = 187.80, BR only = 280.83, Incremental (Bedaquiline + BR vs. BR) = −93.04
						Per patient DALYs lost	Years	Bedaquiline + BR = 9.21, BR only = 13.78, Incremental (Bedaquiline + BR vs. BR) = −4.56
						Incremental cost per QALY gained	GBP	Bedaquiline + BR Dominates (−£10,008.75)
						Incremental cost per DALY avoided	GBP	Bedaquiline + BR Dominates (−£2504.95)
	Kong et al. 2023 [35] (no data for the study conducted year)	Bloodstream infection	Carbapenem-resistant *Klebsiella pneumoniae*	1. Ceftazidime-avibactam (CAZ-AVI) 2. Polymyxin B (PMB)	Human/Hospital-based	Total Cost	USD	CAZ-AVI = 23,261,700.00, PMB = 23,053,300.00, PMB-based regimen = 25,480,000.00
						QALYs	Years	CAZ-AVI = 1240, PMB = 890, PMB-based regimen = 1180
						Incremental cost	USD	PMB = 208,400.00, PMB-based regimen = −2218,300.00
						Incremental QALYs	Years	PMB = 350, PMB-based regimen = 60
						ICER ($/QALY)	Ratio	PMB = 591.7, PMB-based regimen = −36,730.9 (Dominated)
	Mullins et al. 2006 [36] (2002–2003)	Nosocomial pneumonia	Methicillin-resistant *Staphylococcus aureus*	Linezolid vs. Vancomycin	Human/Hospital-based	Treatment cost (per day)—median	USD	Linezolid = 2888.00, Vancomycin = 2993.00
						Total hospitalization cost	USD	Linezolid = 32,636.00, Vancomycin = 32,024.00
						ICER for linezolid per life saved	USD	3600.00
						Mean treatment durations	Days	Linezolid = 11.3, Vancomycin = 10.7
	Jansen et al. 2009 [30] (2006)	Complicated intraabdominal infections (IAI)	No data	Ertapenem vs. Piperacillin/Tazobactam	Human/Hospital-based	Overall savings per patient (95% uncertainty interval)	Euro	355.00 (480.00–1205.00)
						Cost savings with ertapenem—estimated increase (95% uncertainty interval)	Euro	672.00 (232.00–1617.00)
						QALYs (95% uncertainty interval)	Years	0.17 (0.07–0.30)
	Fawsitt et al. 2020 [37] (2017–2018)	Genotype 1 noncirrhotic treatment-naive patients	Hepatitis C Virus (HPV)	Nonstructural protein 5A (NS5A)	Human/Hospital-based	Incremental net monetary benefit (INMB)	GBP	Baseline testing vs. standard 12 weeks of therapy = 11,838.00
								Shortened 8 weeks of treatment (no testing) = 12,294.00
	Sado et al. 2021 [44] (2008–2013)	Pharmacotherapy-resistant depression	No data	Cognitive behavioral therapy (CBT) vs. Treatment as usual (TAU)	Human/Hospital-based	ICER (CBT vs. TAU)	USD (per QALY)	All patients (*n* = 73): −142,184.00 1.2. Moderate/severe patients (*n* = 50): 18,863.00
						Incremental costs (95% CI)	USD	All patients (*n* = 73): 905.00 (408.00–1394.00)
							USD	Moderate/severe patients (*n* = 50): 785.00 (267.00–1346.00)
	Cara et al. 2018 [55] (2011–2014)	Nosocomial pneumonia due to multidrug-resistant Gram-negative bacteria	Gram-negative pathogen	Low- (LDC) vs. High-dose colistin (HDC)	Human/Hospital-based	Average total direct costs per episode of colistin treatment	Saudi Arabian Riyal (SAR)	LDC: 24,718.42
								HDC: 27,775.25
						Incremental cost (per nephrotoxicity avoided)	SAR	3056.28
	Weinstein et al. 2001 [23] (No data for the study conducted year)	Genotypic resistance—HIV infection	No data	No data	Human/Community-based	CPCRA trial—Cost of no genotypic antiretroviral resistance testing	USD	90,360.00
						CPCRA trial—Cost of Genotypic antiretroviral resistance testing	USD	93,650.00
						VIRADAPT trial—Cost of no genotypic antiretroviral resistance testing	USD	91,980.00
						VIRADAPT trial—Cost of Genotypic antiretroviral resistance testing	USD	97,790.00
						CPCRA trial—Cost-effectiveness ratio of Genotypic antiretroviral resistance testing	USD/QALY gained	17,900.00
						VIRADAPT trial—Cost-effectiveness ratio of Genotypic antiretroviral resistance testing	USD/QALY gained	16,300.00
	Barbieri et al. 2005 [121] (2000)	Rheumatoid arthritis	No data	Infliximab plus methotrexate (MTX) vs. MTX alone	Human/Community-based	Primary analysis (Incremental cost, Incremental QALYs, and Incremental cost/QALYs, respectively)	GBP, Years, and Ratio, respectively	8576.00, 0.26, 33,618.00
						Radiographic progression analysis (Incremental cost, Incremental QALYs, and Incremental cost/QALYs, respectively)		7835.00, 1.53, 5111.00
						Intent-to-treat analysis (Incremental cost, Incremental QALYs, and Incremental cost/QALYs, respectively)		14,635.00, 0.40, 36,616.00
						Lifetime analysis (Incremental cost, Incremental QALYs, and Incremental cost/QALYs, respectively)		30,147.00, 1.26, 23,936.00
	Marseille et al. 2020 [46] (2004–2017)	Chronic treatment-resistant posttraumatic stress disorder	No data	1. Methylenedioxy methamphetamine (MDMA) 2. MDMA-assisted psychotherapy (MAP)	Human/Hospital-based	Net costs (1 year)	USD	7,608,691.00
						QALYs	Years	288
						Cost per QALY gained	USD	26,427.00
						MAP intervention cost		7543.00
						QALYs savings	Years	2517
	Mac et al. 2019 [47] (2017)	No data	Vancomycin-resistant *Enterococci*	No data	Human/Hospital-based	Incremental QALYs for VRE screening and isolation	Years	0.0142
						Incremental cost for VRE screening and isolation	Canadian dollars	112.00
						ICER	Canadian dollars/per QALY	7850.00
	Wassenberg et al. 2010 [22] (2001–2004)	Nosocomial infections	Methicillin-resistant *Staphylococcus aureus*	No data	Human/Hospital-based	0% of Attributable mortality	Life years gained, discounted (in the replacement scenario)—Years	0
						10% of Attributable mortality		7.0
						20% of Attributable mortality		15.7
						30% of Attributable mortality		26.8
						40% of Attributable mortality		41.7
						50% of Attributable mortality		62.3
						0% of Attributable mortality	Cost per life year gained per year MRSA policy (in the replacement scenario)—Euro	Not applicable
						10% of Attributable mortality		45,912.00
						20% of Attributable mortality		21,357.00
						30% of Attributable mortality		13,165.00
						40% of Attributable mortality		9062.00
						50% of Attributable mortality		6590.00
	Papaefthymiou et al., 2019 [63] (2012–2016)	*Helicobacter pylori* infection	*Helicobacter pylori*	1. 10-day concomitant use of (a) pantoprazole or (b) esomeprazole 2. 10-day sequential use of (c) pantoprazole or (d) esomeprazole 3. 14-day hybrid using esomeprazole.	Human/Hospital-based	Cost-effectiveness analysis ratio (CEAR)—10-day concomitant regimen with esomeprazole (Lowest)	Euro	179.17
						CEAR—10-day concomitant regimen with pantoprazole		183.27
						CEAR—Hybrid regimen (Higher)		187.42
						CEAR—10-day sequential use of pantoprazole		204.12
						CEAR—10-day sequential use of esomeprazole		216.02
	Bhavnani et al. 2009 [122] (2002–2005)	No data	Methicillin-resistant *Staphylococcus aureus*	Daptomycin vs. Vancomycin-gentamicin	Human/Hospital-based	Stratum I—for daptomycin	USD	4082.00 (1062.00–13,893.00)
						Stratum I—for vancomycin-gentamicin		560.00 (66.00–1649.00)
						Stratum II—for daptomycin		4582.00 (1109.00–21,882.00)
						Stratum II—for vancomycin-gentamicin		1635.00 (163.00–33,444.00)
						Stratum III—for daptomycin		23,639.00 (6225.00–141,132.00)
						Stratum III—for vancomycin-gentamicin		26,073.00 (5349.00–187,287.00)
	Hollinghurst et al. 2014 [39] (2008–2010)	Treatment-resistant depression	No data	Cognitive–behavioral therapy (CBT)	Human/Hospital-based	Mean cost of CBT per participant	GBP	910.00
						Incremental cost-effectiveness ratio	GBP	14,911.00
	Martin et al. 2007 [38] (2006)	Community-acquired pneumonia	1. *S. pneumoniae* 2. *H. influenzae* 3. Atypicals (fluoroquinolones and macrolides) 4. Atypicals (b-lactams)	1. Moxifloxacin 2. Coamoxiclav 3. Clarithromycin 4. Azithromycin 5. Doxycycline 6. Roxithromycin 7. Amoxicillin 8. Cefuroxime axetil	Human/Community-based	Moxifloxacin/coamoxiclav	Total cost in France—Euro	161.40
						Clarithromycin/moxifloxacin		276.56
						Coamoxiclav/clarithromycin		278.64
						Moxifloxacin/coamoxiclav	Total cost in USA —USD	719.86
						Azithromycin/moxifloxacin		876.33
						Coamoxiclav/azithromycin		881.94
						Doxycycline/azithromycin		908.01
						Moxifloxacin/coamoxiclav	Total cost in Germany—Euro	240.60
						Roxithromycin/moxifloxacin		250.59
						Amoxicillin/roxithromycin		268.91
						Cefuroxime axetil/moxifloxacin		314.33
	Xiridou et al., 2016 [26] (2016)	Gonococcal infections	*Neisseria gonorrheae*	Dual therapy (Ceftriaxone and Azithromycin) vs. Monotherapy (Ceftriaxone)	Human/Hospital-based	Annual cost	Euro	112,853.00
						Annual QALYs	Years	0.000174
						Annual ICER	Ratio	1.91 × 10^8^
						Cumulative costs	Euro	1,355,146.00
						Cumulative QALYs	Years	0.000495
						Cumulative ICER	Ratio	9.74 × 10^8^
						Resistance—monotherapy	Percentage	0.01
						Resistance—Dual therapy	Percentage	0 (Zero)
	Liao et al., 2019 [51] (2019)	Metastatic breast cancer	No data	Utidelone plus Capecitabine vs. Capecitabine alone	Human/Community-based	Incremental Cost	USD	13,370.25
						QALYs	Years	0.1961
						ICER per QALY	USD	68,180.78
	Machado et al., 2005 [60] (2005)	Ventilation-associated nosocomial pneumonia	Methicillin-resistant *Staphylococcus aureus*	Linezolid vs. Vancomycin	Human/Hospital-based	Vancomycin	The per unit cost —USD	47.73
						Generic vancomycin		14.45
						Linezolid		214.04
						Linezolid	Efficacy in VAP-MRSA—Percentage	62.2
						Vancomycin		21.2
						Vancomycin	The total cost per cured patient—USD	13,231.65
						Generic vancomycin		11,277.59
						Linezolid		7764.72
	Xin et al., 2020 [58] (2020)	1. Platinum-resistant recurrent 2. Metastatic head and neck squamous cell carcinoma	No data	Pembrolizumab vs. Standard-of-care (SOC)	Human/Hospital-based	Total mean cost—Pembrolizumab	USD	45,861.00
						Total mean cost—SOC	USD	41,950.00
						QALYs gained—Pembrolizumab	Years	0.31
						QALYs gained—SOC	Years	0.25
						ICER	Ratio [USD]	65,186.00/QALY
	Wan et al., 2016 [27] (2016)	Nosocomial pneumonia	Methicillin-resistant *Staphylococcus aureus*	Linezolid vs. Vancomycin	Human/Hospital-based	Beijing—Treatment cost (linezolid vs. vancomycin)	Chinese Yuan	79,551.00 (95% CI ¼ 72,421.00–86,680.00) vs. 77,587.00 (70,656.00–84,519.00)
						Beijing—ICER (linezolid over vancomycin)	Ratio (Chinese Yuan)	19,719.00 (143,553.00 to 320,980.00)
						Guangzhou—Treatment cost (linezolid vs. vancomycin)	Chinese Yuan	90,995.00 (82,598.00–99,393.00) vs. 89,448.00 (81,295.00–97,601.00)
						Guangzhou—ICER (linezolid over vancomycin)	Ratio (Chinese Yuan)	15,532.00 (185,411.00 to 349,693.00)
						Nanjing—Treatment cost (linezolid vs. vancomycin)	Chinese Yuan	82,383.00 (74,956.00–89,810.00) vs. 80,799.00 (73,545.00–88,054.00)
						Nanjing—ICER (linezolid over vancomycin)	Ratio (Chinese Yuan)	15,904.00 (161,935.00 to 314,987.00)
						Xi’an—Treatment cost (linezolid vs. vancomycin)	Chinese Yuan	59,413.00 (54,366.00–64,460.00) vs. 57,804.00 (52,613.00–62,996.00)
						Xi’an—ICER (linezolid over vancomycin)	Ratio (Chinese Yuan)	16,145.00 (100,738.00 to 234,412.00)
	Liu et al., 2021 [45] (2021)	Chronic hepatitis C virus genotype 1b infection (HCV GT 1b)—Nonstructural protein 5A (NS5A) resistance	No data	No data	Human/Hospital-based	Overall increase in total healthcare cost	USD	13.50
						Overall increase in QALYs	Years	0.002
						ICER	Ratio (USD/QALY)	6750/QALY gained
	Breuer and Graham, 1999 [61] (1999)	*Helicobacter pylori* Infection	*Helicobacter pylori*	Endoscopy plus biopsy followed by empirical antibiotic treatment of *H. pylori*-positive ulcer patients.	Human/Setting—No data	Cost saving per 1000 patients treated (Treatment A)	USD	37,000.00
						Additional cost per 1000 patients treated		8027.00
	Niederman et al., 2014 [29] (2014)	Nosocomial pneumonia	Methicillin-resistant *Staphylococcus aureus*	1. Vancomycin 2. Linezolid	Human/Hospital-based	Total treatment cost for patients without renal failure	USD	44,176.00
						Total treatment cost for patients with renal failure		52,247.00
						Total treatment cost difference (for patients who developed renal failure compared with those who did not)		8000.00
						Cost-effectiveness ratio (for linezolid compared with vancomycin)—per treatment success		16,516.00
	Lowery et al., 2013 [123] (2013)	Recurrent platinum-resistant ovarian cancer	No data	Routine care vs. routine care plus early referral to a palliative medicine specialist (EPC)	Human/Hospital-based	Cost savings (associated with EPC)	USD	1285.00
						ICER	Ratio: USD/QALY	37,440.00/QALY
						Base case mean cost (EPC group)	USD	5017.00
						Base case mean cost (routine group)	USD	6303.00
	Chappell et al., 2016 [124] (2016)	Platinum-resistant ovarian cancer	No data	1. Bevacizumab (BEV)—Bevacizumab 10 mg/kg biweekly dose 2. Chemotherapy	Human/Hospital-based	Cost (CHEMO)	USD	21,611.00
						Cost (CHEMO + BEV)	USD	66,511.00
						Progression-free survival (CHEMO)	Months	3.4
						Progression-free survival (CHEMO + BEV)	Months	6.7
						ICER	Ratio [Cost/progression-free life-year saved (USD/PF-LYS)]	160,000.00
				1. Bevacizumab (BEV) -Bevacizumab 15 mg/kg once-every-3-week dose 2. Chemotherapy	Human/Hospital-based	Cost (CHEMO)	USD	18,857.00
						Cost (CHEMO + BEV)	USD	48,861.00
						Progression-free survival (CHEMO)	Months	3.4
						Progression-free survival (CHEMO + BEV)	Months	6.7
						ICER	Ratio (USD/PF-LYS)	100,000.00
	Reed et al., 2009 [52] (2009)	Metastatic breast cancer (Progressing after anthracycline and taxane treatment)	No data	Ixabepilone Plus Capecitabine	Human/Hospital-based	For patients receiving ixabepilone plus capecitabine	USD	60,900.00
						For patients receiving capecitabine alone		30,000.00
						The estimated life expectancy gain with ixabepilone	Months	1.96 (95% CI, 1.36–2.64)
						The estimated gain in quality-adjusted survival	Months	1.06 (95% CI, 0.09–2.03)
						The incremental cost-effectiveness ratio per QALY	USD	359,000.00 (95% CI, 183,000.00–4,030,000.00)
	Ross and Soeteman et al., 2020 [40] (2020)	Treatment-resistant depression	No data	Esketamine Nasal Spray	Human/Hospital-based	QALY gained	Years	0.07
						Healthcare cost	USD	16,995.00
						Base case ICER—Societal	Ratio (USD/QALY)	237,111.00/QALY
						Base case ICER—healthcare sector	Ratio (USD/QALY)	242,496.00/QALY
	Simpson et al., 2009 [59] (2009)	Pharmacotherapy-resistant major depression	No data	Antidepressant therapy—Transcranial magnetic stimulation (TMS)	Human/Hospital-based	Cost per treatment session—sham treatment	USD	300.00
						ICER associated with TMS		34, 999.00/per QALY
						ICER (including productivity gains due to clinical recovery)		6667.00/per QALY
						Net cost savings (associated with TMS		1123.00 per QALY
						Net cost savings (including productivity losses)		7621.00
	Phillips et al., 2023 [53] (2023)	No data	Multidrug-resistant *Salmonella typhi*	Typhoid conjugate vaccines (TCVs)—When an outbreak occurs over the 10-year time horizon	Human/Community-based	Expected net cost for reactive vaccination (routine + campaign)	USD	1.1. 111,213.00
						Expected net cost for no vaccination (base case)		1.2. 128,360.00
						Expected net cost for preventative vaccination (routine + campaign)		131,749.00
						Expected net cost for preventative vaccination (routine only)		154,148.00
				Typhoid conjugate vaccines (TCVs)—When no outbreak occurs (preoutbreak incidence)	Human/Community-based	Expected net cost for no vaccination (base case)	USD	5893.00
						Expected net cost for preventative vaccination (routine only)		26,704.00
						Expected net cost for preventative vaccination (routine + campaign)		27,109.00
				Typhoid conjugate vaccines (TCVs)—When an outbreak has already occurred (postoutbreak incidence)	Human/Community-based	Expected net cost for no vaccination (base case)	USD	7242.00
						Expected net cost for preventive vaccination (routine + campaign)		49,406.00
						Expected net cost for preventative vaccination (routine only)		51,717.00
	Cock et al., 2009 [24] (2009)	Nosocomial pneumonia	Suspected methicillin-resistant *Staphylococcus aureus*	Linezolid vs. Vancomycin	Human/Hospital-based	Average total costs per episode (linezolid vs. vancomycin)	Euro	12,829.00 vs. 12,409.00
						Death rate (linezolid vs. vancomycin)	Percentage	20.7 vs. 33.9
						Life-years gained (linezolid vs. vancomycin)	Years	14.0 life-years vs. 11.7 life-years
						Incremental costs (linezolid vs. vancomycin)	Euro	3171.00 vs. 4813.00
						Mean length of stay	Days	11.2 vs. 10.8 days
	Lynch et al., 2011 [64] (2011)	Selective serotonin reuptake inhibitor-resistant depression	Combined cognitive behavior therapy (CBT) and medication switch vs. medication switch only	Combined Therapy vs. Medication	Human/Hospital-based	Additional depression-free days (DFDs)	Days	8.3
						DFD QALYs	Years	0.020
						Depression-improvement days (DIDs)	Days	11.0
						Cost per DFD/ICER	USD	188.00
	Gordon et al. 2023 [49] (2021)	1. Complicated intra-abdominal infection 2. Complicated urinary tract infection 3. Hospital-acquired pneumonia 4. Ventilator-associated pneumonia	Drug-resistant Gram-negative pathogens (*Escherichia coli*, *Klebsiella pneumoniae*, and *Pseudomonas aeruginosa*)	1. Piperacillin/tazobactam 2. Meropenem	Human/Hospital-based	Hospital length of stay—Reduction in AMR levels (10%)	Days	509,568
						Hospitalization costs—Reduction in AMR levels (10%)		593,282,404.00
						Life-years lost—Reduction in AMR levels (10%)		27,093
						QALYs lost—Reduction in AMR levels (10%)		23,876
						Hospital length of stay—Reduction in AMR levels (20%)	AUD	508,617
						Hospitalization costs—Reduction in AMR levels (20%)		592,173,849.00
						Life-years lost—Reduction in AMR levels (20%)		29,575
						QALYs lost—Reduction in AMR levels (20%)		22,903
						Hospital length of stay—Reduction in AMR levels (50%)	Years	505,762
						Hospitalization costs—Reduction in AMR levels (50%)		588,847,600.00
						Life-years lost—Reduction in AMR levels (50%)		22,692
						QALYs lost—Reduction in AMR levels (50%)		20,045
						Hospital length of stay—Reduction in AMR levels (95%)	Years	501,479
						Hospitalization costs—Reduction in AMR levels (95%)		583,856,587.00
						Life-years lost—Reduction in AMR levels (95%)		17,972
						QALYs lost—Reduction in AMR levels (95%)		15,396
	Rosu et al., 2023 [50] (2023)	Rifampicin-resistant tuberculosis	No data	1. Bedaquiline Oral regimen (Oral) 2. An injectable drug containing Bedaquiline (6-month) 3. 9-month injectable drug containing Bedaquiline (Control)	Human/Hospital-based	Total provider cost—Ethiopia (oral/6 months/control)	USD	3378.10/2549.00/2876.60
						Total Provider cost—India (oral/6 months/control)		1628.00/1374.70/1422.10
						Total Provider cost—Moldova (oral/control)		3362.90/3128.90
						Total Provider cost—Uganda (oral/control)		5437.90/4712.50
						Total participant cost—Ethiopia (oral/6 months/control)		2247.80/893.70/1586.90
						Total participant cost—India (oral/6 months/control)		1415.70/1293.60/1427.80
						Total participant cost—Moldova (oral/control)		7059.10/11,658.60
						Total participant cost—Uganda (oral/control)		2176.20/2787.50
						Total societal cost—Ethiopia (Oral/6 months/Control)		5625.90/3442.70/4463.50
						Total societal cost—India (Oral/6 months/Control)		3079.70/2668.00/2849.90
						Total societal cost—Moldova (Oral/Control)		10,422.00/14,787.50
						Total societal cost—Uganda (Oral/Control)		7614.10/7500.00
						QALYs—Ethiopia (Oral/6 months/Control)	Years	0.8981/0.9002/0.9050
						QALYs—India (Oral/6 months/Control)		0.7439/0.7932/0.7644
						QALYs—Moldova (Oral/Control)		0.9627/0.9235
						QALYs—Uganda (Oral/Control)		0.6937/0.7343
	Matsumoto et al., 2021 [31] (2021)	1. Complicated urinary tract infections 2. Complicated intra-abdominal infections 3. Hospital-associated pneumonia, including ventilator-associated pneumonia	Drug-resistant Gram-negative pathogen: *Escherichia coli*, *Klebsiella pneumoniae*, and *Pseudomonas aeruginosa*	Piperacillin/tazobactam (first-line treatment) or meropenem (second-line treatment)	Human/Community-based	Life year savings	Years	4249,096
						QALY savings	Years	3,602,311
						Bed days savings	Days	4,422,284
						Saved hospitalization cost	Japan Yen (USD)	117.6 billion (1.1 billion)
						Net monitory benefit	Japan Yen	18.1 trillion (169.8 billion)
COI	McCollum et al. 2007 [70] (2003)	Complicated Skin and soft-tissue infections (cSSTIs)	Methicillin-resistant *Staphylococcus aureus*	1. Treatment I = Intravenous/Oral Linezolid 2. Treatment II = Intravenous Vancomycin	Human/Hospital-based	Hospitalization cost	USD	Treatment I: 4510.00
								Treatment II: 6478.00
						Total cost	USD	Treatment I: 6009.00
								Treatment II: 7329.00
						Lenth of stay	Days	Treatment I: 6.8
								Treatment II: 10.3
						Cure rate of MRSA group	Percentage	Treatment I: 80.0
								Treatment II: 71.4%
						Length of stay reduction (Treatment I vs. II)	Days	3.5
						Duration of intravenous treatment	Days	Treatment I: 1.4
								Treatment II: 10.9
						Duration of intravenous treatment reduction (Treatment I vs. II)	Days	9.5
	Touat et al. 2019 [76] (2015)	13 Acute infections ^b^	Nine pathogens ^c^	No data	Human/Hospital-based	Total cost	Euro	109.3 million
						Cost per stay (mean)	Euro	1103.00
						Excess length of stay (mean)	Days	1.6
	Wely et al. 2004 [87] (1998–2001)	Infertility	Clomiphene citrate-resistant polycystic ovary syndrome	Laparoscopic electrocautery strategy (Treatment I) vs. Ovulation induction-Recombinant FSH (Treatment II)	Human/Hospital-based	Direct cost (mean)	Euro	Treatment I and II: 4664. and 5418.00
						Indirect cost (mean)		Treatment I and II: 644.00 and 507.00
						Total cost (mean)		Treatment I and II: 5308.00 and 5925.00
	Patel et al. 2014 [86] (2012)	Nosocomial pneumonia	Methicillin-resistant *Staphylococcus aureus*	Linezolid (Treatment I) vs. Vancomycin (Treatment II)	Human/Hospital-based	Direct cost (medical and drug)	Euro	Treatment I and II: 15,116.00 and 15,239.00
						ICER [favored Treatment I (VS Treatment II)]		123.00
						Total cost per successfully treated patient		Treatment I and II: 24,039.00 and 25,318.00
	Roberts et al. 2021 [81] (2019)	No data	1. *Streptococcus pneumoniae* 2. *S. aureus* 3. *Salmonella* spp. 4. *Escherichia coli* 5. *K. pneumoniae* 6. *Acinetobacter* spp.	No data	Human/Setting—no data	Cost of establishing a laboratory (for capacity of 10,000 specimens) per year	USD (Range)	254,000.00–660,000.00
						Cost for laboratory processing (100,000 specimens) per year		394,000.00–887,000.00
						Cost per specimen		22.00–31.00
						The cost per isolate (10,000 specimens)		215.00—304.00
						The cost per isolate (100,000 specimens)		105.00–122.00
	Song et al. 2022 [72] (2017)	Multidrug-resistant organisms’ bacteremia	1. Methicillin-resistant *Staphylococcus aureus* (MRSA) 2. Vancomycin-resistant *Enterococci* (VRE) 3. Multidrug-resistant *Acinetobacter baumannii* (MRAB) 4. Multidrug-resistant *pseudomonas aeruginosa* (MRPA) 5. Carbapenem-resistant *Enterobacteriaceae* (CRE) 6. *Methicillin-susceptible S.aureus* (MSSA) 7. Non-Multidrug-resistant *A. baumannii* (Non-MDR ABA) 8. Non-Multidrug-resistant *P. aureus* (Non-MDR PAE) 9. Susceptible Enterobacteriaceae 10. Susceptible *Enterococcus*	No data	Human/Hospital-based (tertiary)	Reported cases (MRSA, MRAB, MRPA, CRE, and VRE)	Number of patients	260, 87, 18, 20, and 101
						90-day mortality rates (MRSA, MRAB, MRPA, CRE, and VRE)	Percentage	30.4, 63.2, 16.7, 55.0, and 47.5
						Additional costs caused by bacteremia (MRSA, MRAB, MRPA, CRE, and VRE)	USD	15,768.00, 35,682.00, 39,908.00, 72,051.00, and 33,662.00
						Estimated bacteremia cases (annual)/(MRSA, MRAB, MRPA, CRE, and VRE)	Number of patients	4070, 1396, 218, 461, and 1834
						Estimated deaths (annual)/(MRSA, MRAB, MRPA, CRE, and VRE)	Number of deaths	1237, 882, 36, 254, and 871
						Estimated cost (annual)/(MRSA, MRAB, MRPA, CRE, and VRE)	USD	84,707,359.00, 74,387,364.00, 10,344,370.00, 45,850,215.00, and 79,215,694.00
						Estimated total cases (annual)/(for all 5 resistant pathogens)	Number of patients	7979
						Estimated total deaths (annual)/(for all 5 resistant pathogens)	Number of deaths	3280
						Estimated total cost (annual)/(for all 5 resistant pathogens)	USD	294,505,002.00
						Length of stay (MRSA, MSSA, MRAB, Non-MDR ABA, MRPA, Non-MDR PAE, CRE, Susceptible *Enterobacteriaceae*, VRE, Susceptible *Enterococcus*)	Days	29.8, 28.8, 30.2, 27.9, 41.6, 27.3, 55.4, 21.3, 48.2, 41.3
						Hospital cost (MRSA, MSSA, MRAB, Non-MDR ABA, MRPA, Non-MDR PAE, CRE, Susceptible *Enterobacteriaceae*, VRE, Susceptible *Enterococcus*)	USD	16,386.00, 15,297.00, 24,452.00, 16,946.00, 26,168.00, 17,447.00, 73,248.00, 14,998.00, 47,779.00, 33,365.00
						LOS difference (MRSA vs. MSSA, MRAB vs. Non-MDR ABA, MRPA vs. Non-MDR PAE, CRE vs. Susceptible *Enterobacteriaceae*, VRE vs. Susceptible *Enterococcus*)	Days	1.0, 2.3, 14.3, 34.1, 6.8
						Hospital cost difference (MRSA vs. MSSA, MRAB vs. Non-MDR ABA, MRPA vs. Non-MDR PAE, CRE vs. Susceptible *Enterobacteriaceae*, VRE vs. Susceptible *Enterococcus*)	USD	1089.00, 7507.00, 8721.00, 58,250.00, 14,414.00
	Zhen et al. 2020a [65] (2013–2015)	No data	1. Carbapenem-resistant *Klebsiella pneumoniae* (CRKP) 2. *Pseudomonas aeruginosa* (CRPA) 3. *Acinetobacter baumannii* (CRAB) 4. Carbapenem-susceptible *K. pneumoniae* (CSKP) 5. Carbapenem-susceptible *P. aeruginosa* (CSPA) 6. Carbapenem-susceptible *A. baumannii* (CSAB)	No data	Human/Hospital-based (tertiary)	Total Hospital Cost (CRKP vs. CSKP, CRPA vs. CSPA, and CRAB vs. CSAB)	USD	14,252.00, 4605.00, and 7277.00
						Length of stay (CRKP vs. CSKP, CRPA vs. CSPA, and CRAB vs. CSAB)	Days	13.2, 5.4, and 15.8
						Hospital mortality rate	Percentage difference	Between CRKP and carbapenem-susceptible *K. pneumoniae* (CSKP) = 2.94%
								Between CRAB and carbapenem-susceptible *A. baumannii* (CSAB) = 4.03
								Between CRPA and Carbapenem-susceptible *P. aeruginosa* (CSPA) = 2.03
	Zhen et al. 2020b [89] (2013–2015)	No data	1. Third-generation cephalosporin-resistant *E. coli* 2. Third-generation cephalosporin-susceptible *E. coli* 3. Third-generation cephalosporin-resistant *K. pneumoniae* 4. Third-generation cephalosporin-susceptible *K. pneumoniae*	No data	Human/Hospital-based (tertiary)	Total hospital cost [Excluding length of stay (LOS) before culture]—Median: (3GCSEC, 3GCREC, 3GCSKP, and 3GCRKP)	USD	3867.00, 5233.00, 8084.00, and 15,754.00
						Total hospital cost (Including LOS before culture)–Median: (3GCSEC, 3GCREC, 3GCSKP, and 3GCRKP)	USD	4057.00, 5197.00, 9699.00, and 14,463.00
						LOS (excluding LOS before culture	Days	16, 20, 20, 31
						LOS (including LOS before culture)	Days	17, 19.5, 23, 30
						Mortality rate (excluding LOS before culture)	Percentage	2.15, 2.7, 3.65, 6.74
						Mortality rate (including LOS before culture)	Percentage	2.16, 2.49, 3.81, 6.51
						Total hospital cost difference (excluding LOS before culture)	USD	3GCREC vs. 3GCSEC = 1366.00
								3GCRKP vs. 3GCSKP = 7671.00
						Total hospital cost difference (including LOS before culture)	USD	3GCREC vs. 3GCSEC = 1140.00
								3GCRKP vs. 3GCSKP = 4763.00
						Total LOS difference (excluding LOS before culture)	Days	3GCREC vs. 3GCSEC = 4
								3GCRKP vs. 3GCSKP = 11
						Total LOS difference (including LOS before culture)	Days	3GCREC vs. 3GCSEC = 2.5
								3GCRKP vs. 3GCSKP = 7
						Mortality rate difference (excluding LOS before culture)	Percentage	3GCREC vs. 3GCSEC = 0.55
								3GCRKP vs. 3GCSKP = 3.09
						Mortality rate difference (including LOS before culture)	Percentage	3GCREC vs. 3GCSEC = 0.33
								3GCRKP vs. 3GCSKP = 2.7
	Girgis et al. 1995 [125] (No data for the study conducted year)	No data	Multidrug-resistant *Salmonella typhi septicemia*	1. Cefixime 2. Ceftriaxone 3. Aztreonam	Human/Hospital-based	Drug cost (Per day)—(Cefixime, Ceftriaxone, and Aztreonam)	Egyptian pounds	22.00, 100.00, and 200.00
						Drug delivery cost (Per day)—(Cefixime, Ceftriaxone, and Aztreonam)		2. 2.00, 4.00, and 12.00
						Hospital stay cost (Per day)—(Cefixime, Ceftriaxone, and Aztreonam)		3. 68.00, 68.00, and 68.00
						Total cost (Per day)—(Cefixime, Ceftriaxone, and Aztreonam)		4. 92.00, 172.00, and 280.00
						Drug cost (Total)—(Cefixime, Ceftriaxone, and Aztreonam)		5. 308.00, 500.00, and 1400.00
						Drug delivery cost (Total)—(Cefixime, Ceftriaxone, and Aztreonam)		6. 28.00, 20.00, and 84.00
						Hospital stay cost (Total)—(Cefixime, Ceftriaxone, and Aztreonam)		7. 952.00, 340.00, and 476.00
						Total cost (Total)—(Cefixime, Ceftriaxone, and Aztreonam)		8. 1288.00, 860.00, and 1960.00
	Rijt et al. 2018 [77] (2011–2016)	No data	Methicillin-resistant *Staphylococcus aureus*	No data	Human/Hospital-based	Cost per patient per day (median)	Euro	83.80
						Cost per patient per day (Range)		16.89–1820.09
	Naylor et al. 2020 [67] (2017)	Bloodstream infection	Antibiotic-resistant *Staphylococcus aureus* bloodstream infections	1. Oxacillin 2. Gentamicin 3. Cephalosporins 4. Carbapenems 5. Fluroquinolones 6. Penicillins	Human/Hospital-based	Excess cost	International Dollars (2017)	Per infection = 6392.00
								Per infection associated with Oxacillin resistance = 8155.00
								Per infection associated with Gentamicin resistance = 5675.00
						Excess length of stay	Days	SA BSI = 11.6
								SA BSI resistant to 1st generation Cephalosporins = 13.7
								SA BSI resistant to Carbapenems = 13.7
								SA BSI resistant to Gentamicin = 10.3
								SA BSI resistant to Fluroquinolones = 11.6
								SA BSI resistant to Penicillins = 12.9
								SA BSI resistant to Oxacillin = 14.8
	Browne et al. 2016 [68] (2012)	No data	Methicillin-resistant *Staphylococcus aureus* bacteremia-infective endocarditis	1. Daptomycin 2. Vancomycin	Human/Hospital-based	Drug cost (per patient)	GBP	Daptomycin = 3404.00
								Vancomycin = 1991.00
								Incremental costs = 1413.00
						Monitoring cost (per patient)	GBP	Daptomycin = 226.00
								Vancomycin = 372.00
								Incremental costs = −146.00
						Hospital cost (per patient)	GBP	Daptomycin = 14,252.00
								Vancomycin = 14,796.00
								Incremental costs = −544.00
						Adverse events cost (per patient)	GBP	Daptomycin = 34.00
								Vancomycin = 6.00
								Incremental costs = 28.00
						Total cost (per patient)	GBP	Daptomycin = 17,917.00
								Vancomycin = 17,165.00
								Incremental costs = 752.00
	Zhen et al. 2021 [88] (2013–2015)	No data	Seven pathogens ^d^	No data	Human/Hospital-based	Total hospital cost—Susceptible (mean)	USD	9558.00
						Total hospital cost—Single-drug resistance (SDR) (mean)		10,702.00
						Total hospital cost—Mean difference (Susceptible vs. SDR)		1144.00
						Total hospital cost—multidrug resistance (MDR)		13,017.00
						Total hospital cost—Mean difference (Susceptible vs. MDR)		3391.00
						Length of hospital stay—Susceptible (mean)	Days	22.01
						Length of hospital stay—Single-drug resistance (SDR) (mean)		26.07
						Length of hospital stay—Mean difference (Susceptible vs. SDR)		4.09
						Length of hospital stay—multidrug resistance (MDR)		27.7
						Length of hospital stay—Mean difference (Susceptible vs. MDR)		5.48
						In-hospital mortality rates—Susceptible (mean)	Percentage	1.92
						In-hospital mortality rates—Single-drug resistance (SDR) (mean)		2.67
						In-hospital mortality rates—Mean difference (Susceptible vs. SDR)		0.78
						In-hospital mortality rates—multidrug resistance (MDR)		3.58
						In-hospital mortality rates—Mean difference (Susceptible vs. MDR)		1.50
	Liu et al. 2022 [82] (2017–2018)	Healthcare-associated infections	No data	No data	Human/Hospital-based (tertiary)	The additional total medical expenses (for HAIs)	USD	164.63
						Medical expenses (for HAIs)		114.96
						Out-of-pocket expenses (for HAIs)		150.79
						Hospitalization days (for HAIs)	Days	7
						The additional total medical expenses (for HAIs AMR)	USD	381.15
						Medical expenses (for HAIs AMR))		202.37
						Out-of-pocket expenses (for HAIs AMR		370.56
						Hospitalization days (for HAIs AMR)	Days	9
	Labreche et al. 2013 [79] (2011)	Skin and soft tissue infections	Methicillin-resistant *Staphylococcus aureus*	1. Bacitracin ointment 2. Mupirocin ointment 3. Cephalexin 500 mg capsules 4. Ciprofloxacin 500-mg tablets 5. Clindamycin 300 mg capsules 6. Clindamycin 150 mg capsules 7. Doxycycline 100 mg capsules 8. Trimethoprim–sulfamethoxazole double-strength tablets 9. Clindamycin 600 mg intravenous solution	Human/Community-based	Estimated Additional Costs of Treatment Failure—Hospital admission	USD	1. 17,591.07
						Estimated Additional Costs of Treatment Failure—Outpatient incision and drainage		2. 2130.96
						Estimated Additional Costs of Treatment Failure—Emergency department visit		3. 754.84
						Estimated Additional Costs of Treatment Failure—Mean additional cost (per patient)		4. 1933.71
						Unit cost of antibiotics—Bacitracin ointment		5.23/tube
						Unit cost of antibiotics—Mupirocin ointment		0.31/25 g
						Unit cost of antibiotics—Cephalexin 500 mg capsules		0.11/capsule
						Unit cost of antibiotics—Ciprofloxacin 500 mg tablets		0.15/tablet
						Unit cost of antibiotics—Clindamycin 300 mg capsules		0.20/capsule
						Unit cost of antibiotics—Clindamycin 150 mg capsules		0.07/capsule
						Unit cost of antibiotics—Doxycycline 100 mg capsules		0.04/capsule
						Unit cost of antibiotics—Trimethoprim–sulfamethoxazole double-strength tablets		0.04/tablet
						Unit cost of antibiotics—Clindamycin 600 mg intravenous solution		2.24/600 mg
	Kim et al. 2001 [6] (1996–1998)	No data	Methicillin-resistant *Staphylococcus aureus*	No data	Human/Hospital-based (tertiary)	Additional hospital days attributable to MRSA infection (mean)	Days	14
						Total attributable cost to treat MRSA infections	Canadian Dollar (CAD)	287,200.00
						Per patient-attributable cost to treat MRSA infections		14,360.00
						The total cost for isolation and management of colonized patients		128,095.00
						Per admission cost for isolation and management of colonized patients		1363.00
						Costs for MRSA screening in the hospital		109,813.00
						Costs associated with MRSA in Canadian hospitals (Range)		42,000,000.00–59,000,000.00
	Uematsu et al. 2016 [66] (2013)	Pneumonia	Methicillin-resistant *Staphylococcus aureus*	No data	Human/Hospitals and Community based	Median (IQR) length of stay (MRSA group and Control)	Days	21 (14–33) and 14 (9–23)
						Median (IQR) antibiotic cost (MRSA group and Control)	USD	757.00 (436.00–1482.00) and 152.00 (77.00–347.00)
						Median hospitalization cost (MRSA group and Control)		8751.00 (6007.00–14,598.00) and 4474.00 (3214.00–6703.00)
						Length of stay (Propensity score-matched group)	Days	9 (1.6)
						Antibiotic cost (Propensity score-matched group)	USD	1044.00 (101.00)
						Hospitalization cost (Propensity score-matched group)		5548.00 (580.00)
	Vasudevan et al. 2015 [78] (2007–2011)	Systemic inflammatory response syndrome	Multidrug-resistant Gram-negative bacilli	No data	Human/Hospital-based (tertiary)	Median total hospitalization costs (range) for patients with RGNB	Singapore Dollars	2637.80 (458.70–20,610.30)
						Median total hospitalization costs (range) for patients with no GNB		2795.90 (506.90–4882.30)
	Esther et al. 2012 [84] (2007–2009)	Multidrug resistance in Gram-negative infections	1. *A. baumannii* 2. *Escherichia coli* 3. *Klebsiella pneumoniae* 4. *Proteus mirabilis* 5. *P. aeruginosa* 6. *Enterobacter* spp.	No data	Human/Hospital-based	Total hospitalization cost (IQR)	Singapore Dollars	22,651.24 (11,046.84–48,782.93)
						Excess l hospitalization cost		8638.58
	Roberts et al. 2009 [73] (2000)	Antimicrobial-resistant infection	No data	No data	Human/Hospital-based	Medical costs attributable to ARI (Range)	USD	18,588.00–29,069.00
						Excess duration of hospital stays (Range)	Days	6.4–12.7
						Societal costs (Range)	USD	10,700,000.00–15,000,000.00
						Total cost	USD	13,350,000.00
	Nahuis et al. 2012 [126] (1998–2001)	No data	Clomiphene citrate-resistant polycystic ovary syndrome	1. Laparoscopic electrocautery of the ovaries 2. Ovulation induction with gonadotrophins	Human/Hospital-based	Mean costs per first live birth—for the electrocautery group	Euro	11,176.00 (95% CI: 9689.00–12,549.00)
						Mean costs per first live birth—for the recombinant FSH group		14,423.00 (95% CI: 12,239.00–16,606.00)
						Mean difference—Electrocautery vs. Recombinant FSH group		3247.00 (95% CI: 650.00–5814.00)
	Wozniak et al. 2019 [7] (2014)	1. *E. coli* bloodstream infection 2. *E. coli* urinary tract infection 3. *K. pneumoniae* bloodstream infection 4. *K. pneumoniae* urinary tract infection 5. *P. aeruginosa* bloodstream infection 6. *P. aeruginosa* respiratory tract infection 7. *E. faecium* bloodstream infection 8. *S. aureus*—bloodstream infection 9. *S. aureus* respiratory tract infection	1. *Escherichia coli* 2. *Klebsiella pneumoniae* 3. *Pseudomonas aeruginosa* 4. *Enterococcus faecium* 5. *Staphylococcus aureus* infection	1. Gentamicin or Ceftriaxone 2. Meropenem 3. Vancomycin 4. Linezolid 5. Flucloxacillin	Human/Hospital-based	Total cost (95% Uncertainty interval)	Australian dollar	Ceftriaxone-resistant *E. coli* BSI: 5839,782.00 (2,288,318.00–11,176,790.00)
								Ceftriaxone-resistant KP BSI: 1,351,360.00 (358,717.00–3,158,370.00)
								Ceftazidime-resistant PA BSI: 108,581.00 (48,551.00–202,756.00)
								Ceftazidime-resistant PA RTI: 1,296,324.00 (456,198.00–2,577,397.00)
								VRE BSI: 1404,064.00 (415,766.00–3,287,542.00)
								MRSA BSI: 5,546,854.00 (339,633.00 –22,688,754.00)
								MRSA RTI: 1,525,552.00 (726,903.00–2,791,453.00)
	Young et al. 2007 [127] (2004–2005)	Multidrug-resistant *Acinetobacter baumannii* infection	*Acinetobacter baumannii*	No data	Human/Hospital-based	Attributable excess patient charge due to *Acinetobacter baumannii*	USD	60,913.00
						Excess hospital days due to *Acinetobacter baumannii*	Days	13
						Mean hospital charge (Case vs. Control)	USD	306,877.00 vs. 135,986.00
						Mean duration of hospitalization (Case vs. Control)	Days	25.4 vs. 7.6
	Madan et al. 2020 [69] (2012–2018)	Multidrug-resistant tuberculosis	No data	No data	Human/Hospital-based	Healthcare costs per participant (South Africa—Long-term treatment)	USD	8340.70
						Healthcare costs per participant (South Africa—short-term treatment)		6618.00
						Healthcare costs per participant (South Africa—short-term treatment)		6096.60
						Healthcare costs per participant (Ethiopia—Long-term treatment)		4552.30
						Medication cost savings—South Africa	Percentage (USD)	67.0 (1157.00 of total 1722.80)
						Medication cost savings—Ethiopia		35.0 (545.20 of 1544.30)
	Zhen et al. 2018 [71] (2014–2015)	Multiple drug-resistant intra-abdominal infections	No data	No data	Human/Hospital-based	Total medical cost (TMC)—among patients with MDR pathogens	Chinese Yuan	131,801.00
						Total medical cost—among patients without MDR pathogen		90,201.00
						Sum of all attributable total medical costs (for whole country)		37,000,000,000.00
						The societal costs		111,000,000,000.00
						The mean TMC difference between the MDR and non-MDR groups		90,201.00
	Desai et al. 2021 [75] (2019)	Treatment-resistant depression	No data	Esketamine nasal spray + oral antidepressant (ESK + oral AD) vs. Oral AD plus nasal placebo (oral AD + PBO)	Human/Setting—no data	Commercial	USD	85,808.00 vs. 100,198.00
						Medicaid		76,236.00 vs. 96,067.00
						Veteran’s Affairs		77,765.00 vs. 104,519.00
						Integrated Delivery Network		103,924.00 vs. 142,766.00
	Szukis et al., 2021 [85] (2014–2018)	Major depressive disorders—MDD (psychosis, schizophrenia, manic/bipolar disorder, or dementia) with and without treatment-resistant depression	No data	No data	Human/Hospital-based	Incidence rate ratio—IRR in non-TRD MDD	Ratio	1.7 [95% CI 1.57–1.83]
						Incidence rate ratio (IRR)—Non-MDD		5.04 [95% CI 4.51–5.63]
						Mean difference in total healthcare cost (TRD and non-TRD MDD)	USD	5906.00
						Mean difference in total healthcare cost (TRD and non-MDD)		11,873.00
	Stewardson et al., 2016 [74] (2016)	Bloodstream infections	1. Cephalosporin-resistant *Enterobacteriaceae* 2. Methicillin-susceptible *Staphylococcus aureus* 3. Methicillin-resistant *Staphylococcus aureus*	No data	Human/Hospital-based (tertiary)	Length of stay—3GCRE	Days (95% Confidence Interval)	9.3 (9.2–9.4)
						Length of stay—MSSA		11.5 (11.5–11.6)
						Length of stay—MRSA		13.3 (13.2–13.4)
						Cost from hospital perspective—lowest per infection cost (for 3GCSE): Using economic valuations	Euro (95% Credible Interval)	320.00 (80.00–1300.000
						Cost from hospital perspective—lowest per infection cost (for 3GCSE): Using accounting valuations		4000.00 (2400.00–6700.00)
						Cost from hospital perspective—highest annual cost (With MSSA): Using economic valuation	Euro (95% credible interval)	77,000.00 (19,000.00–300,000.00)
						Cost from hospital perspective—highest annual cost (With MSSA): Using accounting valuations		970,000.00 (590,000.00–1,600,000.00)
	Lester et al., 2023 [128] (2023)	Third-generation cephalosporin-resistant bloodstream infection	1. 3GC-R *E. coli* 2. *Klebsiella* spp.	No data	Human/Hospital-based	Mean health provider cost per participant	USD (95% CR)	110.27 (22.60–197.95)
						Additional indirect cost (Patients with resistant BSI)	USD (95% CR)	155.48 (67.80–378.78)
						Additional direct nonmedical costs (Patients with resistant BSI)	USD (95% CR)	20.98 (36.47–78.42)
	Lu et al., 2021 [129] (2021)	Pneumococcal disease	No data	Pneumococcal conjugate vaccine (PCV) vs. Antibiotics (penicillin, amoxicillin, third-generation cephalosporins, and meropenem)	Human/Hospital-based	Reduction in AMR due to PCV against penicillin, amoxicillin, and third-generation cephalosporins (99% coverage in 5 years)	Percentage	6.6, 10.9 and 9.8
						Reduction in AMR due to PCV against penicillin, amoxicillin, and third-generation cephalosporins (coverage increased to 85% over 2 years, and followed by 3 years to reach 99%)		10.5, 17.0, 15.4
						Reduction in cumulative costs of AMR (including direct and indirect costs of patients and caretakers) (coverage increased to 99% coverage in 5 years)	USD	371 million
						Reduction in cumulative costs of AMR (including direct and indirect costs of patients and caretakers) (Coverage increased to 85% over 2 years)		586 million
	Janis et al., 2014 [83] (2014)	Hand infection	Methicillin-resistant *Staphylococcus aureus*	Vancomycin or Cefazolin	Human/Hospital-based	Mean length of stay—Patients randomized to cefazolin	Days	5.75
						Mean length of stay—Patients randomized to vancomycin		4.23
						Cost of treatment—Patients randomized to cefazolin	USD	6693.23
						Cost of treatment—Patients randomized to vancomycin		4589.41
	Mahmoudi et al., 2020 [80] (2020)	No data	No data	Pre and post Antimicrobial Stewardship Program	Human/Hospital-based	Total defined daily dose per 1000 patient days: Pre-ASP	Number of doses	129.1
						Total defined daily dose per 1000 patient days—post-ASP	Number of doses	95.2
						Total defined daily dose per 1000 patient days—Difference	Percentage	−26.3
						Total monthly cost of antimicrobial administration—Pre-ASP	USD	437,572.00
						Total monthly cost of antimicrobial administration—post-ASP	USD	254,520.00
						Total monthly cost of antimicrobial administration—Difference	Percentage (*p* value)	−41.8 (< 0.001)
	Imai et al., 2022 [130] (2022)	Carbapenem-resistant (CR) bacterial infection—pneumonia, urinary tract infection, biliary infection, and sepsis/Carbapenem-susceptible (CS) infections	No data	Carbapenem	Human/Hospital-based	Cost of CR vs. CS infections—Medications	USD—Median	3477.00 vs. 1609.00
						Cost of CR vs. CS infections—Laboratory tests		2498.00 vs. 1845.00
						Cost of CR vs. CS infections—Hospital stays		14,307.00 vs. 10,560.00
Disease burden	Cassini et al. 2019 [94] (2015)	1. Bloodstream infections 2. Urinary tract infections 3. Respiratory tract infections 4. Surgical site infections 5. Other infections	Seven pathogens ^e^	No data	Human/Community-based	Disability-adjusted life-years (DALYs)	Lost years of full health (Uncertainty intervals)	874,541 (768,837–989,068)
						Attributable deaths	Number of deaths (Uncertainty intervals)	33,110 (28,480–38,430)
						Attributable deaths per 100,000 population	Number of deaths (Uncertainty intervals)	6.44 (5.54–7.48)
						DALYs per 100,000 population	Lost years of full health (Uncertainty intervals)	170 (150–192)
	Kritsotakis et al. 2017 [90] (2012)	1. Lower respiratory tract infections 2. Bloodstream infections	Carbapenem-resistant Gram-negative pathogen	No data	Human/Hospital-based	1. HAI prevalence	1. Percentage (95% CI)	1. 9.1 (7.8–10.6)
						2. Estimated annual HAI incidence	2. Percentage (95% CI)	2. 5.2 (4.4–5.3)
						3. Length of stay (LOS)	3. Days (95% CI)	3. 4.3 (2.4–6.2)
						4. Mean excess LOS	4. Days	4. 20
	Le and Miller. 2001 [93] (2000)	Uncomplicated urinary tract infections	*Escherichia coli* (EC)	1. Trimethoprim–sulfamethoxazole (TMP-SMZ) 2. Fluoroquinolone (FQ)	Human/Hospital-based	The mean cost of TMP-SMZ: When the proportion of resistant EC was 0%	USD	92.00
						The mean cost of TMP-SMZ: When the proportion of resistant EC was 20%		106.00
						The mean cost of TMP-SMZ: When the proportion of resistant EC was 40%		120.00
						Mean cost of empirical FQ treatment		107.00
	Tsuzuki et al., 2021 [91] (2021)	Bloodstream infections	Nine pathogens ^f^	No data	Human/Hospital-based	DALYs (Per 100,000 population)	Years	137.9 [95% uncertainty interval (UI) 130.7–145.2]
						DALYs [Median (IQR)] per 100,000 population		145.7 (108.4–172.4)
	Wozniak et al., 2022 [92] (2022)	1. Bloodstream infection 2. Urinary tract infection 3. Respiratory tract infection	1. *Enterococcus* spp. 2. *E. coli*, *K. pneumoniae* 3. *P. aeruginosa* 4. *S. aureus*	No data	Human/Hospital-based	Cost of premature death	Australian Dollar	438,543,052.00
						Total hospital cost	Australian Dollar	71,988,858.00
						Loss of quality-adjusted life years	Years	27,705
CBA	Puzniak et al. 2004 [95] (1997–1999)	No data	Vancomycin-resistant *Enterococcus* (VRE)	No data	Human/Hospital-based	Attributable annual cost/Incremental cost of gown policy	USD	73,995.00
						Averted cases	No of cases	58
						Total averted VRE attributable medical intensive care unit cost	USD	493,341.00
	Simoens et al. 2009 [96] (2007)	No data	Methicillin-resistant *Staphylococcus aureus*	No data	Human/Hospital-based	Benefits of search and destroy policy (intensive care unit)	Euro	16,058.00
						Benefits of search and destroy policy (Gerontology unit)	Euro	9321.00
						Benefit-cost ratio of search and destroy policy (intensive care unit)	Ratio	1.17
						Benefit-cost ratio of search and destroy policy (Gerontology unit)	Ratio	1.16
CUA	Varon-Vega et al. 2022 [97] (2019)	No data	Carbapenem-resistant *Klebsiella pneumoniae*	Ceftazidime-Avibactam (CAZ-AVI) vs. Colistin-meropenem (COL + MEM)	Human/Hospital-based	Increase in QALYs per patient (CAZ-AVI vs. COL + MEM)	Years	1.76
						Incremental costs (CAZ-AVI vs. COL + MEM)	USD	2521.00
						Incremental cost-effectiveness ratio	USD	3317.00
	Chen et al. 2019 [98] (2018)	Complicated urinary tract infection	Gram-negative Bacteria (GNB)	1. Ceftolozane/Tazobactam 2. Piperacillin/Tazobactam	Human/Hospital-based (tertiary)	Total medical cost	USD	4199.01 vs. 3594.76
						QALYs	Years	4.8 vs. 4.78
						Additional cost per discounted QALY gained	USD	32,521.08
CMA	Farquhar et al. 2004 [99] (1996–1999)	Infertility	Clomiphene citrate-resistant polycystic ovary syndrome	Laparoscopic ovarian diathermy (Treatment I) vs. Gonadotrophins (Treatment II)	Human/Hospital-based (tertiary)	Chance of pregnancy	Percentage	Treatment I and II: 27.0 and 33.3
						Chance of live birth	Percentage	Treatment I and II: 14.0 and 19.0
						Cost per patient	New Zealand dollar (NZD)	Treatment I and II: 2953.00 and 5461.00
						Cost per pregnancy	NZD	Treatment I and II: 10,938.00 and 16,549.00
						Cost per live birth	NZD	Treatment I and II: 21,095.00 and 28,744.00

Note: ^a^ Values are given in this order: AMR POCTs A vs. SC, AMR POCTs B vs. SC, AMR POCTs C vs. SC, AMR POCTs D vs. SC, and AMR POCTs E vs. SC, ^b^ 1. Urinary and genital tract, 2. Devices and prosthesis-related infection, 3. Skin and soft tissues, 4. Lower respiratory tract, 5. Bacteremia and sepsis (alone), 6. Gastrointestinal and Abdominal, 7. Bone and joint, 8. During pregnancy, 9. Heart and mediastinum, 10. Infection in newborns, 11. Ear, nose and throat, 12. Eye, and 13. Nervous system, ^c^ 1. *Escherichia coli*, 2. *Klebsiella,* 3. Other *Enterobacteriaceae,* 4. *S. aureus*, 5. Others *Staphylococcus,* 6. *Pneumococcus*, 7. *Enterococcus*, 8. Other *Streptococcus*, and 9. Gram-negative bacilli, ^d^ 1. *Staphylococcus aureus*, 2. *Enterococcus faecalis*, 3. *Enterococcus faecium*, 4. *Escherichia coli*, 5. *Klebsiella pneumonia*, 6. *Pseudomonas aeruginosa*, and 7. *Acinetobacter baumannii*, ^e^ 1. Colistin-resistant, carbapenem-resistant, or multidrug-resistant *Acinetobacter* spp.; 2. Vancomycin-resistant *Enterococcus faecalis* and *Enterococcus faecium*, 3. Colistin-resistant, carbapenem-resistant, or third-generation cephalosporin-resistant *Escherichia coli*; 4. Colistin-resistant, carbapenem-resistant or third-generation cephalosporin-resistant *Klebsiella pneumoniae*; 5. Colistin-resistant, carbapenem-resistant, or multidrug-resistant *Pseudomonas aeruginosa*; 6. Methicillin-resistant *Staphylococcus aureus*, and 7. Penicillin-resistant and macrolide-resistant *Streptococcus pneumoniae*, ^f^ 1. Methicillin-resistant *Staphylococcus aureus*, 2. Fluoroquinolone-resistant *Escherichia coli*, 3. Third-generation cephalosporin-resistant *E. coli*, 4. Third-generation cephalosporin-resistant *Klebsiella pneumoniae*, 5. Carbapenem-resistant *Pseudomonas aeruginosa*, 6. Penicillin-resistant *Streptococcus pneumoniae*, 7. Carbapenem-resistant Enterobacteriaceae, 8. Vancomycin-resistant Enterococcus, and 9. Multiple drug-resistant *Acinetobacter* spp.
