# Supplementary material for: Existing Evidence from Economic Evaluations of Antimicrobial Resistance—A Systematic Literature Review
Source: Antibiotics (Basel). 2025 Oct 24;14(11):1072. doi: 10.3390/antibiotics14111072 (PMC12649366; doi:10.3390/antibiotics14111072)
Supplement: Supplementary file 1 [file antibiotics-14-01072-s001.zip › Supplementary file S3.pdf]

Supplementary file S3: Data extraction template

Data extraction table template – Part I

Study ID	Author	Citation	Publication year	Study conducted year	Study conducted date/s	Country	Site (province, city, district, municipality)	Region	Income group	x (longitude)	y (latitude)
01											

Data extraction table template – Part II

Infection/ Ill Health Condition	Resistant pathogen	Type of antibiotic/ Drug/ Intervention	Sector	Setting	Type of economic evaluations	Value type	Unit of measurement	Value	Study Methods	Study limitations	Note
01											
