# Supplementary material for: Existing Evidence from Economic Evaluations of Antimicrobial Resistance—A Systematic Literature Review
Source: Antibiotics (Basel). 2025 Oct 24;14(11):1072. doi: 10.3390/antibiotics14111072 (PMC12649366; doi:10.3390/antibiotics14111072)
Supplement: Supplementary file 1 [file antibiotics-14-01072-s001.zip › Supplementary file S4.pdf]

Supplementary file S4: Quality appraisal of eligible studies

Study type	Citation	Screening questions		2.1. Is randomization appropriately performed?	2.2. Are the groups comparable at baseline?	2.3. Are there complete outcome data?	2.4. Are outcome assessors blinded to the intervention provided?	2.5. Did the participants adhere to the assigned intervention?	Total Marks	Percentage
		S1. Are there clear research questions/objective?	S2. Do the collected data allow to address the research questions/ objective?							
Quantitative randomized controlled trials (n=30, 32.3%)	Jansen et al. 2009	Yes	Yes	Yes	Yes	Yes	Yes	Yes	7	100.0
	McCollum et al. 2007	Yes	Yes	Yes	Yes	Yes	Can't tell	Yes	6	85.7
	Farquhar et al. 2004	Yes	Yes	Yes	Yes	Yes	No	Yes	6	85.7
	Wely et al. 2004	Yes	No	No	Yes	Yes	No	Yes	4	57.1
	Wysham et al. 2017	Yes	Can't tell	Yes	Yes	Yes	No	Yes	4	57.1
	Oppong et al. 2016	Yes	Yes	Yes	Yes	Yes	Yes	Yes	7	100.0
	Yi et al. 2019	Yes	Yes	Yes	Yes	Yes	No	Yes	6	85.7
	Kirwin et al. 2019	Yes	Can't tell	Yes	Yes	Yes	No	Yes	5	71.4
	Pollard et al. 2017	No	Yes	Yes	Yes	Yes	Can't tell	Yes	6	85.7
	Tu et al. 2021	Yes	Yes	Yes	Yes	Yes	No	Yes	6	85.7
	Tringale et al. 2018	Yes	Can't tell	Yes	Yes	Yes	Yes	Yes	5	71.4
	Wolfson et al. 2015	Yes	Yes	Yes	Yes	Yes	Can't tell	Yes	6	85.7
	Mullins et al. 2006	Yes	Yes	Yes	Yes	Yes	Yes	Yes	7	100.0
	Sado et al. 2021	Yes	Yes	Yes	Yes	Yes	Yes	Yes	7	100.0
	Weinstein et al. 2017	Yes	Yes	Yes	Yes	Yes	Can't tell	Yes	6	85.7
	Barbieri et al. 2005	Yes	Yes	Yes	Yes	Yes	Yes	Yes	7	100.0
	Marseille et al. 2020	Yes	Yes	Yes	Yes	Yes	Yes	Yes	7	100.0
	Bhavnani et al. 2009	Yes	Yes	Yes	Yes	yes	No	Yes	6	85.7
	Hollinghurst et al. 2014	Yes	Yes	Yes	Yes	Yes	Can't tell	Yes	6	85.7
	Liao et al., 2019	Yes	Yes	Yes	Yes	Yes	Yes	Yes	7	100.0
	Xin et al., 2020	Yes	Yes	Yes	Yes	Yes	Can't tell	Yes	6	85.7
	Wan et al., 2016	Yes	Yes	Yes	Yes	Yes	Yes	Yes	7	100.0
	Niederman et al., 2014	Yes	Yes	Yes	Yes	Yes	Yes	Yes	7	100.0

	Lowery et al., 2013	Yes	Yes	Yes	Yes	Yes	No	Yes	6	85.7
	Chappell et al., 2016	Yes	Can't tell	Can't tell	Yes	Yes	Can't tell	Yes	4	57.1
	Janis et al., 2014	Yes	Yes	Yes	Yes	Yes	Can't tell	Yes	6	85.7
	Ross & Soeteman et al., 2020	Yes	Can't tell	Can't tell	Can't tell	Yes	Can't tell	Yes	3	42.9
	Simpson et al., 2009	Yes	Yes	Yes	Yes	Yes	Yes	Yes	7	100.0
	Phillips et al., 2023	No	Yes	Yes	No	Yes	Yes	Yes	6	85.7
	Cock et al., 2009	Yes	Yes	Yes	Yes	Yes	Yes	Yes	7	100.0
Quantitative non-randomized studies (n=29, 31.2%)	Puzniak et al. 2004	Yes	Yes	Yes	Yes	Yes	Can't tell	Yes	6	85.7
	Martin et al. 2008	Yes	Yes	Can't tell	Yes	Yes	Can't tell	Yes	5	71.4
	Harding-Esch et al. 2020	Yes	Yes	Yes	Yes	Yes	Can't tell	Yes	6	85.7
	Patel et al. 2014	Yes	Yes	No	Yes	Yes	Can't tell	Yes	5	71.4
	Girgis et al. 1995	Yes	Yes	Yes	Yes	Yes	Yes	Yes	7	100.0
	Brownea et al. 2016	Yes	Yes	Yes	Yes	Yes	Can't tell	Yes	6	85.7
	Cassini et al. 2019	Yes	Yes	Yes	Yes	Yes	Can't tell	Yes	6	85.7
	Larsson et al. 2022	Yes	Yes	Yes	Yes	Yes	Can't tell	Yes	6	85.7
	Rao et al. 1988	No	Can't tell	No	Can't tell	Yes	Can't tell	Yes	2	28.6
	Wang et al. 2015	Yes	yes	Yes	Yes	Yes	Can't tell	Yes	6	85.7
	Kong et al. 2023	Yes	Yes	Yes	Yes	Yes	No	Can't tell	5	71.4
	Jansen et al. 2009	Yes	Yes	Yes	Yes	Yes	Can't tell	Can't tell	5	71.4
	Fawsitt et al. 2020	Yes	Yes	Can't tell	Yes	Yes	Can't tell	Yes	5	71.4
	Cara et al. 2018	Yes	Yes	Yes	Yes	Yes	Can't tell	Yes	6	85.7
	Simoens et al. 2009	Yes	Yes	Can't tell	Yes	Yes	Can't tell	Yes	5	71.4
	Mac et al. 2019	Yes	Yes	Yes	Yes	Yes	Can't tell	Yes	6	85.7
	Wassenberg et al. 2010	Yes	Yes	Yes	Yes	Yes	Can't tell	Yes	6	85.7
	Papaefthymiou et al. 2019	Yes	Yes	Yes	Yes	Yes	Can't tell	Yes	6	85.7
	Le and Miller. 2001	Yes	Yes	Can't tell	Yes	Yes	Can't tell	Yes	5	71.4
	Varón-Vega et al. 2022	Yes	Yes	Can't tell	Yes	Yes	Can't tell	Yes	5	71.4
	Martin et al. 2007	Yes	Yes	Can't tell	Yes	Yes	Can't tell	Yes	5	71.4

	Xiridou et al., 2016	Yes	Can't tell	Can't tell	Yes	Yes	Can't tell	Yes	4	57.1
	Machado et al., 2005	Yes	Yes	Can't tell	Yes	Yes	Can't tell	Can't tell	4	57.1
	Liu et al., 2021	Yes	Can't tell	Yes	Yes	Yes	Can't tell	Can't tell	4	57.1
	Breuer and Graham, 1999	Yes	Can't tell	Can't tell	Yes	Yes	Can't tell	Can't tell	3	42.9
	Matsumoto et al., 2021	Yes	Yes	Yes	Yes	Yes	Can't tell	No	5	71.4
	Lu et al., 2021	Yes	Yes	Can't tell	Yes	Yes	Can't tell	Yes	5	71.4
	Reed et al., 2009	Yes	Yes	Can't tell	Yes	Yes	Can't tell	Yes	5	71.4
	Lynch et al., 2011	Yes	Yes	Yes	Yes	Yes	Can't tell	Yes	6	85.7
Quantitative descriptive studies (n=34, 36.5%)	Touat et al. 2019	Yes	Yes	Yes	Yes	Yes	Can't tell	Yes	6	85.7
	Roberts et al. 2021	Yes	Yes	Yes	Yes	Yes	Can't tell	Yes	6	85.7
	Morgans et al. 2022	Yes	Yes	Can't tell	Can't tell	Yes	Can't tell	Yes	4	57.1
	Song et al. 2022	No	Can't tell	Yes	Yes	Yes	Can't tell	Yes	4	57.1
	Zhen et al. 2020	Yes	Yes	Yes	Yes	Yes	Can't tell	Yes	6	85.7
	Zhen et al. 2020	Yes	Yes	Can't tell	Can't tell	Yes	Can't tell	Yes	4	57.1
	Rijt et al. 2018	Yes	Yes	Yes	Can't tell	Yes	Can't tell	Yes	5	71.4
	Naylor et al. 2020	Yes	Yes	Yes	Yes	Yes	Can't tell	Yes	6	85.7
	Zhen et al. 2021	Yes	Yes	Yes	Yes	Yes	Can't tell	Yes	6	85.7
	Evans et al. 2007	No	Can't tell	Can't tell	Yes	Yes	Can't tell	Yes	3	42.9
	Liu et al. 2022	Yes	Yes	Yes	Yes	Yes	Can't tell	Yes	6	85.7
	Labreche et al. 2013	Yes	Yes	Yes	Yes	Yes	Can't tell	Yes	6	85.7
	Kim et al. 2014	Yes	Yes	Yes	No	Yes	Can't tell	Yes	5	71.4
	Uematsu et al. 2016	Yes	Yes	Yes	Yes	Yes	Can't tell	Yes	6	85.7
	Vasudevan et al. 2015	Yes	Yes	Yes	Yes	Yes	Can't tell	Yes	6	85.7
	Gordon et al. 2023	Yes	Yes	Can't tell	Can't tell	Yes	Can't tell	Yes	4	57.1
	Esther et al. 2012	Yes	Yes	Yes	Yes	Yes	Can't tell	Yes	6	85.7
	Roberts et al. 2009	Yes	Yes	Yes	Yes	Yes	Can't tell	Yes	6	85.7
	Nahuis et al. 2012	Yes	Yes	Yes	Yes	Yes	Can't tell	Yes	6	85.7
	Wozniak et al. 2019	Yes	Yes	Can't tell	Can't tell	Yes	Can't tell	Yes	4	57.1

	Kritsotakis et al. 2017	Yes	Yes	Yes	Yes	Yes	Can't tell	Yes	6	85.7
	Young et al. 2007	Yes	Yes	Yes	No	Yes	Can't tell	Yes	5	71.4
	Madan et al. 2020	Yes	Yes	Can't tell	Can't tell	Yes	Can't tell	Yes	4	57.1
	Zhen et al. 2018	Yes	Yes	Yes	Yes	Yes	Can't tell	Yes	6	85.7
	Desai et al. 2021	Yes	Yes	Can't tell	Can't tell	Yes	Yes	Yes	5	71.4
	Chen et al. 2018	Yes	Can't tell	Can't tell	Can't tell	Yes	Can't tell	Yes	3	42.9
	Szukis et al., 2021	Yes	Yes	Yes	Yes	Yes	Yes	Yes	7	100.0
	Stewardson et al., 2016	Yes	Yes	Yes	Yes	Yes	Yes	Yes	7	100.0
	Rebecca Lester et al., 2023	Yes	Yes	Yes	Yes	Yes	Yes	Yes	7	100.0
	Rosu et al., 2023	No	Yes	No	No	Yes	No	Yes	3	42.9
	Tsuzuki et al., 2021	Yes	Yes	Yes	Yes	Yes	Yes	Yes	7	100.0
	Wozniak et al., 2022	Yes	Yes	Yes	Yes	Yes	Yes	Yes	7	100.0
	Mahmoudi et al., 2020	Yes	Yes	Yes	Yes	Yes	Yes	Yes	7	100.0
	Imai et al., 2022	Yes	Yes	Yes	Yes	Yes	Yes	Yes	7	100.0
