# Supplementary material for: Existing Evidence from Economic Evaluations of Antimicrobial Resistance—A Systematic Literature Review
Source: Antibiotics (Basel). 2025 Oct 24;14(11):1072. doi: 10.3390/antibiotics14111072 (PMC12649366; doi:10.3390/antibiotics14111072)
Supplement: Supplementary file 1 [file antibiotics-14-01072-s001.zip › Supplementary file S5.pdf]

Supplementary file S5: Revised types of economic evaluations

Citation	Reported type of economic evaluation in the article	Changed type of economic evaluation
Matsumoto et al., 2021	Economic and health/clinical burden	CEA
Morgans et al. 2022	Economic evaluation/modelling	CEA
Kirwin et al. 2019	Economic evaluation/modelling	CEA
Evans et al. 2007	Economic evaluation/modelling	CEA
Niederman et al., 2014	Economic evaluation/modelling	CEA
Stewardson et al., 2016	Economic and health/clinical burden	COI
Rebecca Lester et al., 2023	Economic and health/clinical burden	COI
Lu et al., 2021	Economic and health/clinical burden	COI
Imai et al., 2022	Economic and health/clinical burden	COI
Patel et al. 2014	Economic burden	COI
Song et al. 2022	Economic burden	COI
Zhen et al. 2020	Economic burden	COI
Zhen et al. 2020	Economic burden	COI
Zhen et al. 2021	Economic burden	COI
Uematsu et al. 2016	Economic burden	COI
Esther et al. 2012	Economic burden	COI
Roberts et al. 2009	Economic burden	COI
Wozniak et al. 2019	Economic burden	COI
Szukis et al., 2021	Economic burden	COI
Mahmoudi et al., 2020	Economic burden	COI
Nahuis et al. 2012	Economic evaluation/modelling	COI
Young et al. 2007	Economic evaluation/modelling	COI
Madan et al. 2020	Economic evaluation/modelling	COI
Rosu et al., 2023	Economic evaluation/modelling	CEA
Le and Miller. 2001	Decision and cost analysis	Disease burden

Note: CBA: cost-benefit analysis, CEA: cost-effectiveness analysis, COI: cost of illness analysis
