# Supplementary material for: Existing Evidence from Economic Evaluations of Antimicrobial Resistance—A Systematic Literature Review
Source: Antibiotics (Basel). 2025 Oct 24;14(11):1072. doi: 10.3390/antibiotics14111072 (PMC12649366; doi:10.3390/antibiotics14111072)
Supplement: Supplementary file 1 [file antibiotics-14-01072-s001.zip › Supplementary file S6.pdf]

Supplementary file S6: AMR Economic evaluation by income classification

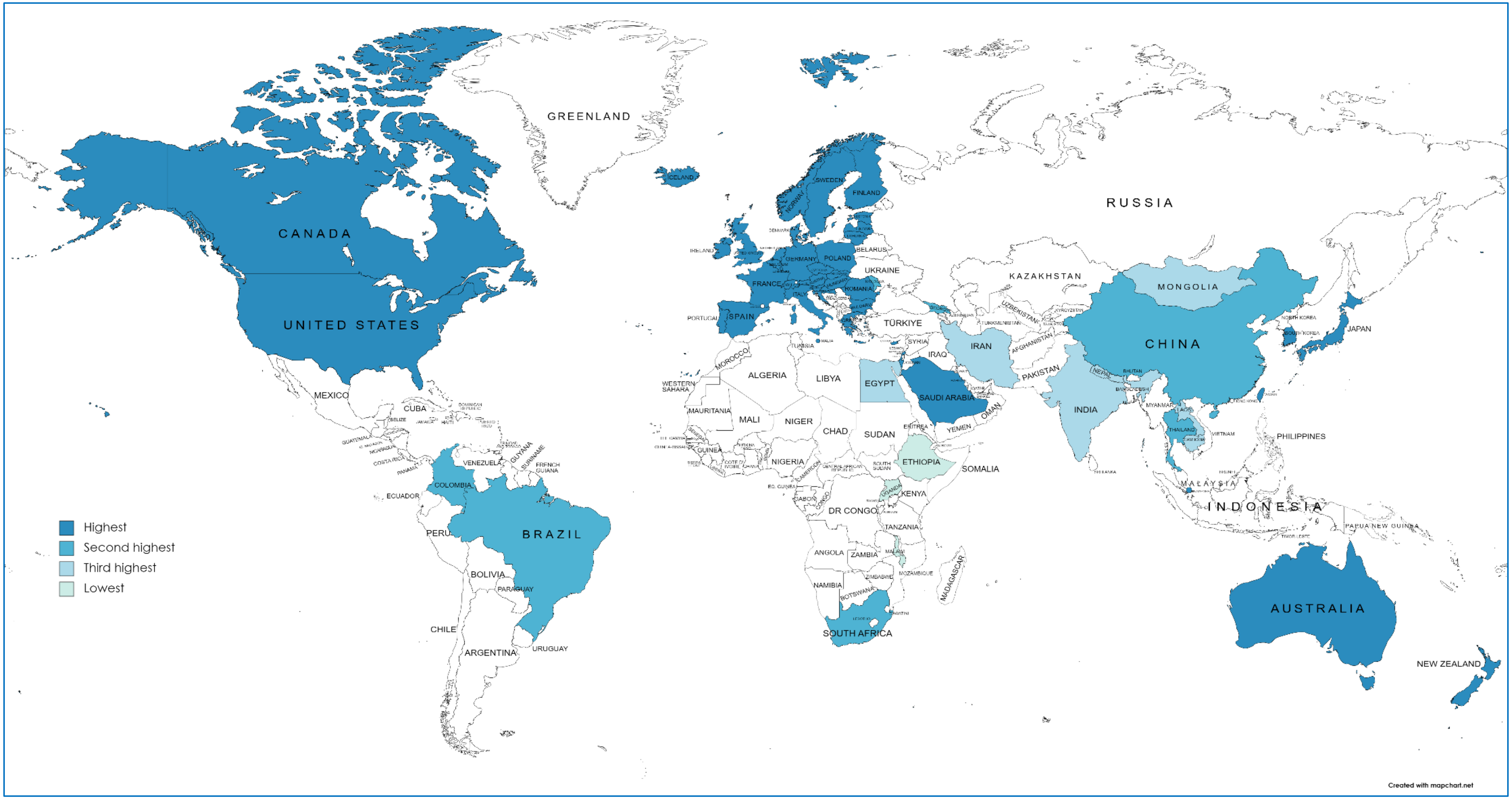

Note: Highest: 131 analysis in 41 HICs (USA, UK, Netherlands, Germany, Japan, France, Canada, Greece, Australia, Sweden, Singapore, Republic of Korea, New Zealand, Taiwan, Saudi Arabia, Belgium, Italy, Spain, Switzerland, Austria, Poland, Portugal, Slovakia, Slovenia, Bulgaria, Croatia, Cyprus, Czech Republic, Denmark, Estonia, Finland, Hungary, Iceland, Ireland, Israel, Latvia, Lithuania, Luxembourg, Malta, Norway, and Romania); second highest: 19 analysis in 7 upper-middle-income countries (China, South Africa, Thailand, Georgia, Moldova, Brazil, and Colombia); third highest: 7 analysis in 7 lower-middle-income countries (Cambodia, Lao People's Democratic Republic (PDR), Mongolia, India, Nepal, Egypt, and Iran); lowest: 5 analysis in 3 low-income countries (Malawi, Ethiopia, and Uganda)
