# Supplementary material for: Existing Evidence from Economic Evaluations of Antimicrobial Resistance—A Systematic Literature Review
Source: Antibiotics (Basel). 2025 Oct 24;14(11):1072. doi: 10.3390/antibiotics14111072 (PMC12649366; doi:10.3390/antibiotics14111072)
Supplement: Supplementary file 1 [file antibiotics-14-01072-s001.zip › Supplementary file S7.pdf]

Supplementary file S7: AMR Economic evaluation by countries

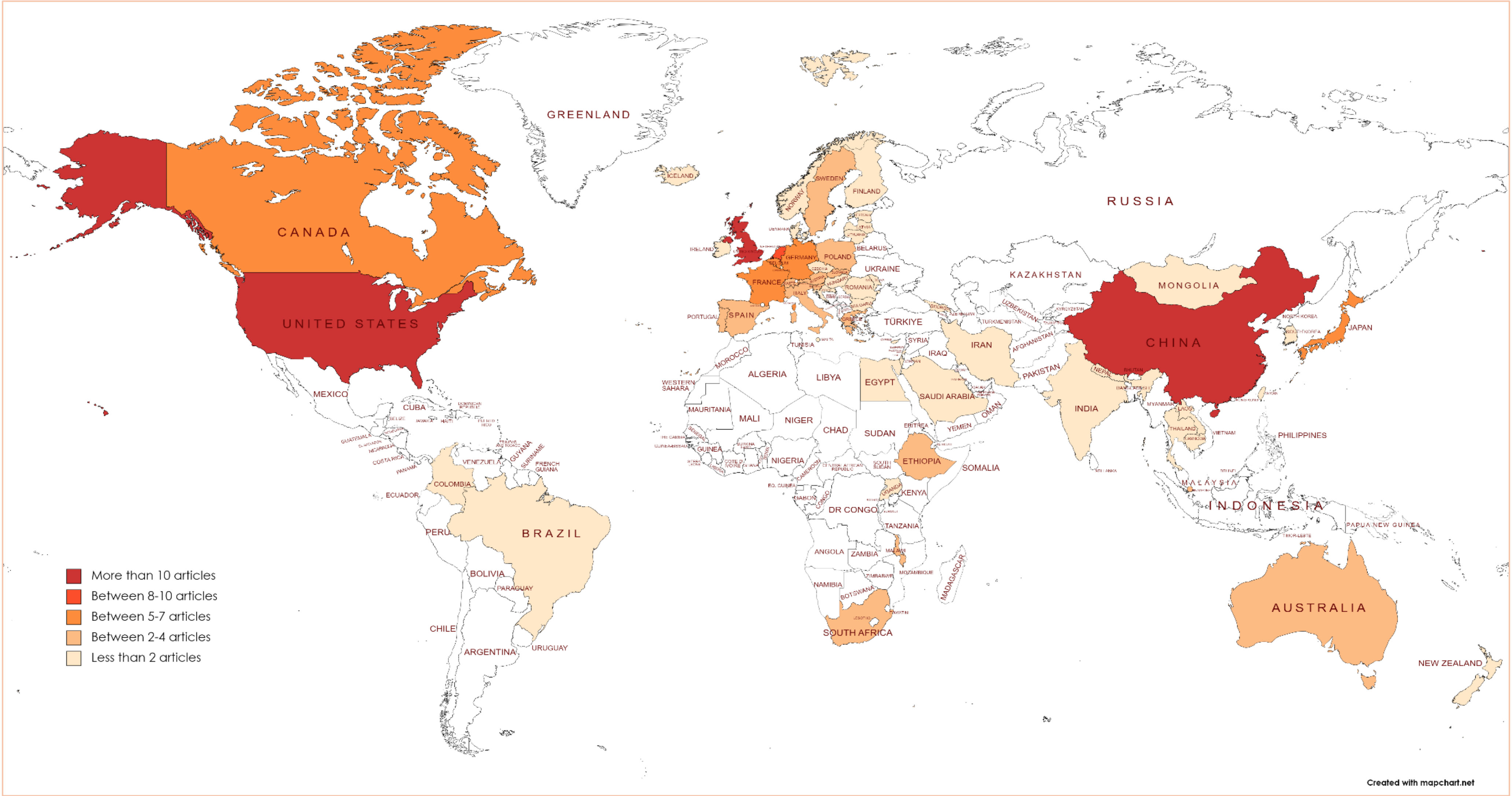

Note: Note: More than 10 articles: USA, China, and UK; 8-10 articles: Netherland; between 5-7 articles: Germany, Japan, France, Canada, and Belgium; 2-4 articles: Italy, Spain, Greece, Australia, Switzerland, Sweden, Singapore, Austria, Poland, Portugal, Slovakia, Slovenia, South Africa, Ethiopia, and Malawi; Less than 2 articles: Cambodia, Lao PDR, Mongolia, Republic of Korea, New Zealand, Taiwan, Thailand, India, Nepal, Georgia, Moldova, Bulgaria, Croatia, Cyprus, Czech Republic, Denmark, Estonia, Finland, Hungary, Iceland, Ireland, Israel, Latvia, Lithuania, Luxembourg, Malta, Norway, Romania, Egypt, Iran, Saudi Arabia, Brazil, Colombia, and Uganda
