# Supplementary material for: Existing Evidence from Economic Evaluations of Antimicrobial Resistance—A Systematic Literature Review
Source: Antibiotics (Basel). 2025 Oct 24;14(11):1072. doi: 10.3390/antibiotics14111072 (PMC12649366; doi:10.3390/antibiotics14111072)
Supplement: Supplementary file 1 [file antibiotics-14-01072-s001.zip › Supplementary file S8.pdf]

Supplementary file S8: Characteristics of selected studies

Study citation	Study conducted year	Country	Income group	WHO region	Type of economic evaluation*
Puzniak et al. 2004	1997-1999	USA	High income	Region of the Americas	CBA
Jansen et al. 2009	2006	UK	High income	European region	CEA
Martin et al. 2008	2006	Belgium	High income	European region	CEA
McCollum et al. 2007	2003	USA	High income	Region of the Americas	COI
Touat et al. 2019	2015	France	High income	European region	COI
Farquhar et al. 2004	1996-1999	New Zealand	High income	Western Pacific region	CMA
Wely et al. 2004	1998-2001	Netherlands	High income	European region	COI
Harding-Esch et al. 2020	2015-2016	UK	High income	European region	CEA
Patel et al. 2014	2012	Germany	High income	European region	COI
Wysham et al. 2017	2015	USA	High income	Region of the Americas	CEA
Roberts et al. 2021	2019	Lao People's Democratic Republic	Lower-middle income	Western Pacific region	COI
		Cambodia	Lower-middle income	Western Pacific region	
		Thailand	Upper-middle income	South-east Asian region	
Morgans et al. 2022	2020	USA	High income	Region of the Americas	CEA
Song et al. 2022	2017	Korea	High income	Western Pacific region	COI
Zhen et al. 2020a	2013-2015	China	Upper-middle income	Western Pacific region	COI
Zhen et al. 2020b	2013-2015	China	Upper-middle income	Western Pacific region	COI
Girgis et al. 1995	No data	Egypt	Lower-middle income	Eastern Mediterranean region	COI
Oppong et al. 2016	2012	Belgium, France, Germany, Italy, Netherlands, Poland, Slovakia, Slovenia, Spain, Sweden, and UK	High income	European region	CEA
Rijt et al. 2018	2011-2016	Netherlands	High income	European region	COI
Naylor et al. 2020	2017	Japan	High income	Western Pacific region	COI
Brownea et al. 2016	2012	UK	High income	European region	COI
Yi et al. 2019	No data	China	Upper-middle income	Western Pacific region	CEA
Cassini et al. 2019	2015	Italy, Greece, Romania, Portugal, Cyprus, France, Slovakia, Poland, Croatia, Hungary, Bulgaria, Malta, Ireland, Slovenia, Czech Republic, Belgium, Spain, UK, Austria, Latvia, Lithuania, Luxembourg, Germany, Denmark, Sweden, Finland, Norway, Netherlands, Estonia, and Iceland	High income	European region	Disease burden
Kirwin et al. 2019	2011-2015	Canada	High income	Region of the Americas	CEA
Zhen et al. 2021	2013-2015	China	Upper-middle income	Western Pacific region	COI
Evans et al. 2007	1996-2000	USA	High income	Region of the Americas	CEA
Pollard et al. 2017	2013	USA	High income	Region of the Americas	CEA

Larsson et al. 2022	2015-2019	Sweden	High income	European region	CEA
Rao et al. 1988	1986-1987	USA	High income	Region of the Americas	CEA
Tu et al. 2021	2019	Canada	High income	Region of the Americas	CEA
Wang et al. 2015	2006-2014	China	Upper-middle income	Western Pacific region	CEA
Tringale et al. 2018	2017	USA	High income	Region of the Americas	CEA
Wolfson et al. 2015	2013	UK	High income	European region	CEA
Kong et al. 2023	No data	China	Upper-middle income	Western Pacific region	CEA
Mullins et al. 2006	2002-2003	USA	High income	Region of the Americas	CEA
Jansen et al. 2009	2006	Netherlands	High income	European region	CEA
Fawsitt et al. 2020	2017-2018	UK	High income	European region	CEA
Sado et al. 2021	2008-2013	Japan	High income	Western Pacific region	CEA
Cara et al. 2018	2016	Saudi Arabia	High income	Eastern Mediterranean region	CEA
Liu et al. 2022	2017-2018	Nepal	Lower-middle income	South-east Asian region	COI
Weinstein et al. 2017	No data	USA	High income	Region of the Americas	CEA
Labreche et al. 2013	2011	USA	High income	Region of the Americas	COI
Barbieri et al. 2005	2000	UK	High income	European region	CEA
Kim et al. 2014	1996-1998	Canada	High income	Region of the Americas	COI
Marseille et al. 2020	2004-2017	USA and Canada	High income	Region of the Americas	CEA
		Switzerland and Israel	High income	European region	
Simoens et al. 2009	2007	Belgium	High income	European region	CBA
Uematsu et al. 2016	2013	Japan	High income	Western Pacific region	COI
Vasudevan et al. 2015	2007-2011	Singapore	High income	Western Pacific region	COI
Mac et al. 2019	2017	Canada	High income	Region of the Americas	CEA
Gordon et al. 2023	2021	Australia	High income	Western Pacific region	CEA
Esther et al. 2012	2007-2009	Singapore	High income	Western Pacific region	COI
Roberts et al. 2009	2000	USA	High income	Region of the Americas	COI
Nahuis et al. 2012	1998-2001	Netherlands	High income	European region	COI
Wassenberg et al. 2010	2001-2004	Netherlands	High income	European region	CEA
Papaefthymiou et al. 2019	2012-2016	Greece	High income	European region	CEA
Wozniak et al. 2019	2014	Australia	High income	Western Pacific region	COI
Kritsotakis et al. 2017	2012	Greece	High income	European region	Disease burden
Le and Miller. 2001	2000	USA	High income	Region of the Americas	Disease burden
Bhavnani et al. 2009	2002-2005	No data	No data	No data	CEA
Young et al. 2007	2004-2005	USA	High income	Region of the Americas	COI
Madan et al. 2020	2012-2018	Ethiopia	Low income	African region	COI
		South Africa	Upper-middle income	African region	
Hollinghurst et al. 2014	2008-2010	UK	High income	European region	CEA

Zhen et al. 2018	2014-2015	China	Upper-middle income	Western Pacific region	COI
Varón-Vega et al. 2022	2019	Colombia	Upper middle income	Region of the Americas	CUA
Desai et al. 2021	2019	USA	High income	Region of the Americas	COI
Martin et al. 2007	2006	France and Germany	High income	European region	CEA
		USA	High income	Region of the Americas	
Chen et al. 2018	2018	Taiwan	High income	Western Pacific region	CUA
Xiridou et al., 2016	2016	Netherlands	High income	European region	CEA
Liao et al., 2019	2019	China	Upper-middle income	Western Pacific region	CEA
Machado et al., 2005	2005	Brazil	Upper-middle income	Region of the Americas	CEA
Xin et al., 2020	2020	China	Upper-middle income	Western Pacific region	CEA
Szukis et al., 2021	2021	USA	High income	Region of the Americas	COI
Wan et al., 2016	2016	China	Upper-middle income	Western Pacific region	CEA
Stewardson et al., 2016	2016	France, Germany, Italy, Switzerland, Spain, and UK	High income	European region	COI
Liu et al., 2021	2021	China	Upper-middle income	Western Pacific region	CEA
Breuer and Graham, 1999	1999	USA	High income	Region of the Americas	CEA
Niederman et al., 2014	2014	USA	High income	Region of Americas	CEA
Lowery et al., 2013	2013	USA	High income	Region of Americas	CEA
Matsumoto et al., 2021	2021	Japan	High income	Region of Americas	CEA
Chappell et al., 2016	2016	Canada	High income	Region of the Americas	CEA
		Austria, Belgium, France, Germany, Greece, Italy, Netherlands, Portugal, Spain, Switzerland, and UK	High income	European region	
Lester et al., 2023	2023	Malawi	Low income	African region	COI
Rosu et al., 2023	2023	Ethiopia and Uganda	Low income	African region	CEA
		South Africa	Upper-middle income	African region	
		India	Lower-middle income	South-east Asian region	
		Moldova and Georgia	Upper-middle income	European region	
		Mongolia	Lower-middle income	Western Pacific region	
Tsuzuki et al., 2021	2021	Japan	High income	Western Pacific region	Disease burden
Wozniak et al., 2022	2022	Australia	High income	Western Pacific region	Disease burden
Lu et al., 2021	2021	China	Upper-middle income	Western Pacific region	COI
Janis et al., 2014	2014	USA	High income	Region of the Americas	COI
Reed et al., 2009	2009	USA	High income	Region of the Americas	CEA
Mahmoudi et al., 2020	2020	Iran	Lowe-middle income	Eastern Mediterranean region	COI
Ross & Soeteman et al., 2020	2020	USA	High income	Region of the Americas	CEA
Simpson et al., 2009	2009	USA	High income	Region of the Americas	CEA
Imai et al., 2022	2022	Japan	High income	Western Pacific region	COI

Phillips et al., 2023	2023	Malawi	Low income	African region	CEA
Cock et al., 2009	2009	Germany	High income	European region	CEA
Lynch et al., 2011	2011	USA	High income	Region of the Americas	CEA

Note: No data denotes that the data for particular interest were unavailable in the original article, *Type of economic evaluation – CBA: cost-benefit analysis, CEA: cost-effectiveness analysis, COI: cost of illness analysis, CUA: Cost-utility analysis, CMA: cost-minimization analysis, *1. Urinary and genital tract, 2. Devices and prosthesis-related infection, 3. Skin and soft tissues 4. Lower respiratory tract 5. Bacteremia and sepsis (alone) 6. Gastrointestinal and abdominal 7. Bone and joint 8. During pregnancy 9. Heart and mediastinum 10. Infection in newborn 11. Ear, nose, and throat 12. Eye 13. Nervous system
